# Context‐dependent effects of a reintroduced ungulate on soil properties are driven by soil texture, moisture, and herbivore activity

**DOI:** 10.1002/ece3.6743

**Published:** 2020-09-07

**Authors:** Vanessa J. Dodge, Valerie T. Eviner, J. Hall Cushman

**Affiliations:** ^1^ Department of Biology Sonoma State University Rohnert Park CA USA; ^2^ Department of Plant Sciences University of California Davis CA USA; ^3^ Department of Natural Resources & Environmental Science University of Nevada Reno NV USA

**Keywords:** context‐dependent effects, ecosystem processes, environmental heterogeneity, long‐term effects, native ungulate herbivore, reintroduction, soil characteristics

## Abstract

Although there is considerable evidence that large mammalian herbivores influence ecosystem‐level processes, studies have reported such widely varying results that generalizations have remained elusive. Here, we use an 18‐year‐old exclosure experiment—stratified across a landscape heterogeneous with respect to soil texture, moisture and herbivore activity—to understand the variable effects of tule elk (*Cervus canadensis nannodes*), a native reintroduced herbivore, on soil properties along the coast of northern California. Elk significantly increased soil bulk density and created a compacted layer at shallow soil depth, while decreasing infiltration rate and pH. The effects of elk on bulk density, penetration resistance, and pH varied with soil type, being least pronounced in coarse, sandy loams, and greatest in loose sand. The effects of elk on nutrient availability varied along gradients of soil texture and moisture. In coarser soils, elk decreased ammonium availability, but increased it in finer soils. Elk also decreased soil moisture content, in part through their positive effect on bulk density, and this effect was most pronounced in coarser soils. Through decreasing soil moisture content, elk also decreased nitrate availability in coarser soils. At greater levels of elk activity (as measured by dung deposition), the elk effect on bulk density was amplified, and this had a corresponding negative effect on nitrate and phosphate availability. Our study has demonstrated that a better understanding of spatial variation in the effects of herbivores on ecosystems can emerge by evaluating their influences across gradients of soil texture, soil moisture, and herbivore activity. These data enabled us to evaluate several frameworks that have been developed to understand the variable effects of herbivores on ecosystems, which is a significant step in reconciling the many competing ideas put forth to explain the context‐dependent effects of large herbivores on grazed ecosystems.

## Graphical abstract

Although there is considerable evidence that large mammalian herbivores influence ecosystem‐level processes, through their effects on vegetation and disturbance regimes, studies have reported such widely varying results that generalizations have remained elusive. Using an 18‐year‐old field experiment, our research evaluates the degree to which physical characteristics of the soil and levels of herbivore activity predict context‐dependent effects of mammalian herbivores on ecosystem‐level processes.

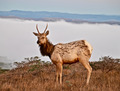

## 1 INTRODUCTION

Through their activities as consumers, disturbance agents, and fertilizers, large herbivores can be major drivers of ecosystem‐level processes throughout many regions of the world (Ruess & McNaughton, 1987; Hobbs & Thompson, 1996; Verchot, Groffman, & Frank, 2002; Binkley, Singer, Kaye, & Rochelle, 2003, see review by Forbes et al., 2019). They can influence a range of soil properties, including nutrient availability and mineralization rates (Bardgett & Wardle, 2003; Hobbs & Thompson, 1996; McNaughton, Banyikwa, & McNaughton, 1997; Pastor, Dewey, Naiman, McInnes, & Cohen, 1993; Ritchie, Tilman, & Knops, 1998), bulk density (Abdel‐Magid, Schuman, & Hart, 1987; Gass & Binkley, 2011; Steffens, Kölbl, Totsche, & Kögel‐Knabner, 2008), infiltration rates (Daryanto, Eldridge, & Wang, 2013; Thrash, 1997), moisture levels (Gass & Binkley, 2011), salinity (Buckeridge & Jefferies, 2007; Chaneton & Lavado, 1996; Lavado & Alconada, 1994), pH (Binkley et al., 2003; Hatton & Smart, 1984), temperature (Bakker, Olff, Olff, Boekhoff, Gleichman, & Berendse, 2004; van der Wal, van Lieshout, & Loonen, 2001), erosion (Ford & Grace, 1998), and microbial communities (Bardgett, Wardle, & Yeates, 1998; Frank, Gehring, Machut, & Phillips, 2003; Murray, Frank, & Gehring, 2010). Some of their ecosystem‐level effects are direct, driven largely by disturbance, and the deposition of metabolic wastes (Frost & Hunter, 2007), whereas others arise indirectly via changes in net primary productivity and the quality and quantity of leaf litter reaching the soil surface (Bardgett & Wardle, 2003; Hobbs & Thompson, 1996). These indirect effects can be driven by changes in plant community composition (Johnson & Cushman, 2007; Ritchie et al., 1998) or can occur independent of them (Schrama, Heijning, et al., 2013).

Although there is considerable evidence that herbivores have major effects on nutrient cycling, usually expressed in terms of nitrogen availability and mineralization, studies have reported widely varying results (Bardgett & Wardle, 2003; Hobbs & Thompson, 1996; McNaughton et al., 1997; Pastor et al., 1993; Ritchie et al., 1998). Some studies have documented that herbivores accelerate nutrient cycling (Augustine & McNaughton, 2006; Frank & Groffman, 1998; Frank, Groffman, Evans, & Tracy, 2000; McNeil & Cushman, 2005; Ruess & McNaughton, 1987; Singer & Schoenecker, 2003), others report that they decelerate nutrient cycling (Augustine & McNaughton, 2006; Bakker, Knops, Milchunas, Ritchie, & Olff, 2009; Gass & Binkley 2011; Pastor et al., 1993; Steffens et al., 2008), and some report no effects (Binkley et al., 2003; Relva, Castan, & Mazzarino, 2014; Vaieretti, Cingolani, Pérez Harguindeguy, & Cabido, 2013). In an effort to understand these variable findings, a number of frameworks have been developed to predict the context‐dependence effects of herbivores on ecosystem processes.

The most commonly cited framework was articulated by Hobbs and Thompson (1996), Ritchie et al. (1998), and Wardle et al. (2004) and uses differences in soil nutrient availability to predict variability in the effects of herbivores on ecosystems. They propose that herbivores will accelerate nutrient cycling in nutrient‐rich ecosystems in two ways: by depositing nutrients in the form of dung and urine, which are more labile than plant litter, and by promoting fast‐growing plant species with high‐quality regrowth, which enhances the quality of leaf litter returned to the soil. Conversely, this framework proposes that herbivores will decelerate nutrient cycling in nutrient‐poor ecosystems by selective herbivory on nutrient‐rich plants, shifting the plant community toward species that produce lower quality leaf litter (Pastor et al., 1993; Pastor & Naiman, 1992; Post & Pastor, 1988).

While this plant‐quality framework explains some of the variation in how herbivores alter ecosystem‐level processes, it fails to account for the full range of effects observed in nature. For instance, contrary to predictions of this framework, several studies have shown that herbivores can decelerate nutrient cycling in nutrient‐rich ecosystems (Bakker et al., 2004; Millett & Edmondson, 2015; Schrama, Heijning, et al., 2013; Stark & Grellmann, 2002; Vaieretti et al., 2013) and increase nitrogen availability in nutrient‐poor sites (Cherif & Loreau, 2013; Sitters, te Beest, Cherif, Giesler, & Olofsson, 2017).

Schrama, Ciska Veen, et al. (2013) have proposed an expansion of the plant‐quality framework to include three key physical characteristics—soil texture, compaction, and moisture content. Other recent studies have also acknowledged the interaction of herbivores with soil physical properties and their impact on soil carbon storage (McSherry & Ritchie, 2013) and grazing lawn formation (Hempson et al., 2014; Veldhuis et al., 2014). Schrama, Ciska Veen, et al. (2013) hypothesize that at intermediate moisture (10%–30% moisture), the plant‐quality framework explains herbivore effects on nitrogen cycling, with changes being closely coupled to changes in plant tissue quality. However, at low and high soil moisture contents, they hypothesize that herbivore‐mediated soil compaction will have a negative effect on nitrogen cycling, particularly on fine‐textured soils. At high moisture sites (>30% moisture), this herbivore‐mediated compaction will drive the water‐logging of soil. At low moisture sites (<10% moisture), compaction will decrease water infiltration and imposes further water limitation. At low moisture on coarse‐textured soils, herbivory can either decrease nitrogen cycling through compaction or increase it through enhancing plant tissue quality. This predictive framework was supported by a study by Schrama, Heijning, et al. (2013) that examined the effects of cattle grazing in a salt marsh across two different soil types. While this study was an important contribution in assessing the framework, a full assessment of the framework's merits requires more studies in diverse systems with different herbivores.

Although not included in Schrama, Ciska Veen, et al. (2013) framework, the intensity with which herbivores use their habitat is another factor that mediates the effects herbivores on nutrient cycling (Ricca, Miles, Van Vuren, & Eviner, 2016; Senft et al., 1987). Both wild and domesticated herbivores exhibit a high level of heterogeneity in activity across a landscape, varying in how much time they spend in a given area and how they use it (Bailey et al., 1996; Homburger, Luscher, Scherer‐Lorenzen, & Schneider, 2015; Senft et al., 1987). Herbivores can serve as vectors for nutrient transfer, moving nutrients from one habitat to another due to seasonal migration (Abbas et al. 2012 Frank, Inouye, Huntly, Minshall, & Anderson, 1994; Frank & McNaughton, 1993; Murray, Webster, & Bump, 2013), or on a day‐to‐day basis, depositing more of their metabolic wastes in areas where they rest and ruminate than where they feed (Abbas et al. 2012; Hobbs 1996; McNaughton, Ruess, & Seagle, 1988; Schonecker, Singer, Zeigenfuss, Binkley, & Menezes, 2004; Singer & Schoenecker, 2003; Seagle 2003).

In this study, we use an 18‐year‐old experiment stratified across a heterogeneous landscape to examine the effects of tule elk (*Cervus canadensis nannodes*), a reintroduced native herbivore, on soil physical and chemical properties along the coast of northern California. Our research addressed the following questions: (a) Does a large, native, mammalian herbivore influence nutrient availability and physical characteristics of the soil, and do these effects vary across a landscape that is heterogeneous in soil texture and moisture? and (b) Is the magnitude of an herbivore's effect on soil characteristics influenced by the intensity with which it uses an area? Our experiment provides a robust approach to assess the generality of Schrama, Ciska Veen, et al. (2013) framework as well as the importance of intensity of herbivore use, by explicitly addressing herbivore effects on ecosystem processes across gradients of herbivore activity, soil textures and moisture levels, and vegetation. For example, while broad categories for soil texture and moisture (coarse/fine; dry/intermediate/ wet), such as those proposed by Schrama, Ciska Veen, et al. (2013), can help shed light on the effects of herbivores on belowground processes, our ability to understand the interactive effects between herbivory and soil texture can be improved by examining these effects of large herbivores on belowground processes along a continuous range of texture and moisture. The heterogeneity within a landscape can also drive spatial differences in the amount of herbivore activity, which can be critical for understanding heterogeneity in the effects of herbivores on belowground processes—a question that cannot be addressed by focusing only on comparisons of areas with versus without herbivores. Addressing these questions will further our understanding of how herbivores influence ecosystem‐level processes and what factors are important to include within a predictive framework.

## 2 METHODS

### 2.1 Study system

Our research was conducted on Tomales Point in Point Reyes National Seashore, approximately 65 km northwest of San Francisco. Bordered by the Pacific Ocean and Tomales Bay, Tomales Point is a 1,030‐ha peninsula that experiences a Mediterranean‐type climate, with moderate rainy winters and cool, foggy summers with very little precipitation. The coastal grasslands on Tomales Point consist of both native and exotic herbaceous plant species interspersed with native shrubs.

Soil maps across our 300‐ha study area identify three distinct soil types as well as a fourth mixed soil type (Kashiwagi, 1985). There are strong correlations between soil types and vegetation type (V. J. Dodge and J. H. Cushman, unpublished data), with each soil type dominated by distinct vegetation: (a) Kehoe variant 138 is a coarse sandy loam (derived from Cretaceous granitic parent rock; Stoffer, ; Wagner, Bortugno, & Kelley, ) characterized by patches of herbaceous vegetation mixed with dense stands of *Baccharis pilularis* (Asteraceae), a long‐lived native shrub (Johnson & Cushman, 2007); (b) Kehoe variant 139 is also a coarse sandy loam (derived from Cretaceous granitic parent rock; Stoffer, ; Wagner et al., ) and is dominated by herbaceous vegetation and largely devoid of shrubs (Johnson & Cushman, 2007); and (c) Sirdrak sand (derived from a Quaternary dune sand parent rock; Stoffer, ; Wagner et al., ) is a loose, structureless sand and is dominated by a short‐lived, native, nitrogen‐fixing shrub, *Lupinus arboreus* (Fabaceae). Sirdrak sand is extremely well drained, resulting in much drier conditions than those seen in either Kehoe soil formation. A mixed soil (hereafter referred to as the mixed K/S soil) occurs along the border of the Kehoe 138 formation and the Sirdrak sand. This soil is heterogeneous and, depending upon sampling location within our exclosure experiment, can exhibit properties of each of the two distinct component soil types, or properties that are a mixture of both of the component soil formations.

Previous research has shown that the four soil formations at this site differ considerably from each other with respect to soil texture, moisture, aboveground plant biomass, and use by elk (Dodge, 2017; Figure S1). In general, the Sirdrak sand and mixed K/S soils were the coarsest, Kehoe 139 soils were the finest, and Kehoe 138 soils fell between these two extremes. Across the study system, soil moisture was negatively correlated with the proportion of coarse material in the soil and accordingly the two coarsest soils (Sirdrak sand and mixed K/S) were significantly drier than the finest soils (Kehoe 139). Aboveground plant biomass varied significantly among the four soil formations, with levels being greatest on the Kehoe 139 formation and least on the Kehoe 138 formation. Elk activity (as estimated by total dung area) also varied significantly among soil formations, being highest on the Kehoe 139 formation and lowest on the Kehoe 138 formation, with intermediate levels on the Sirdrak sand and mixed K/S soil.

Tule elk (*Cervus canadensis nannodes*) is a native ungulate that previously dominated much of coastal and central California. They once numbered 500,000 individuals across their range but hunting and land conversion during the Gold Rush brought them to the brink of extinction by the mid‐1800s (McCullough, 1969). The dramatic decline prompted efforts to protect elk, bolster their numbers, and reintroduce populations to over 20 different sites in California. In 1978, 10 tule elk were reintroduced to a 1,030‐ha wilderness area on Tomales Point. Following their reintroduction, the elk population grew rapidly for two decades, reaching approximately 450 individuals before leveling off. Since 1998, the herd has typically fluctuated between 400 and 600 individuals, although censuses between 2014 and 2016 indicated that the population had declined to fewer than 300 animals, possibly due to prolonged drought (D. Press, unpublished data). The diet of tule elk at Tomales Point consists primarily of herbaceous forbs and grasses, but they also consume shrub foliage during the winter months when there is less herbaceous material available (Gogan & Barrett, 1995).

### 2.2 Exclosure experiment

This study centers around a large‐scale elk exclosure experiment located on Tomales Point in Point Reyes National Seashore. Established by the National Park Service and U.S. Geological Survey in 1998, the experiment consists of 24 36 × 36 m plots distributed across vegetation types and soil formations as described above. Each plot in the experiment is located 350–850 m from the Pacific Ocean. Our 24 plots are organized into 12 pairs, with one plot in each pair randomly assigned fencing to exclude elk and another plot spaced 3 m away and left unfenced to serve as a control. Elevation varies across the study site between approximately 250–400 m, but the fenced and unfenced plots in each pair were located at a similar elevation. Eight plots are on Kehoe variant 138, eight are on Kehoe variant 139, four are on Sirdrak sand, and four are on mixed K/S soil. The fencing that surrounds each exclosure plot is 2.5‐m tall and effectively excludes elk, but not other small‐ or mid‐sized herbivores such as deer or hares (J. H. Cushman, personal observation). Other studies using this exclosure experiment have shown that elk exert major influences on the plant community (Johnson & Cushman, 2007; Lee et al. unpublished data; Richter et al. unpublished data), plant functional traits (Lee et al. unpublished data), invasive exotic grasses (Ender, Christian, & Cushman, 2017), small mammals (Ellis & Cushman, 2018), and ground‐dwelling arthropods (Cecil, Spasojevic, & Cushman, 2019).

### 2.3 Soil physical properties

In order to assess the effects of elk on physical properties of soil, we quantified moisture, soil texture, and bulk density in March of 2015, and measured infiltration rate, and penetration resistance of soil in March of 2016. We collected soil cores from nine equally spaced locations within each plot, avoiding the outer 3‐m edge. Samples were collected using a slide hammer soil core sampler with a 5.1 cm diameter × 5.1 cm depth liner (A.M.S. American Falls, Idaho). We placed soil samples in plastic Ziploc bags, stored them in a cooler for 6 hr, and then transported them back to the laboratory at Sonoma State University, where they were weighed, oven‐dried at 60°C for 72 hr and weighed again. All nine replicate soil samples were analyzed and then averaged, except for soil texture, which was determined on bulked samples. From these samples, we calculated gravimetric moisture and bulk density using the following equations:

$$\text{Gravimetric moisture}=M_{W}‐M_{D}/M_{D}$$

$$\text{Bulk density}\;\left(\rho \right)=M_{D}/V_{S}$$

where *M*_D_ = weight of oven‐dried soil, *M*_W_ = weight of field‐wet soil, and *V*_S_ = volume of soil core. As soils were not rocky or gravelly, stones were not removed from samples prior to determining bulk density (USDA, ).

We quantified soil texture from the dried soil samples by sieving with a standard sieve series (Newark Wire Cloth Company, Clifton, NJ). We weighed each portion of different grain sizes (1–2 mm, 0.5–1 mm, 0.25–0.5 mm, 0.125–0.25 mm, and <0.125 mm) and calculated their proportions of total weight of soil sieved (Kleinhesselink, Magnoli, & Cushman, 2014).

In March of 2016 (during the rainy season of a year that had average rainfall), we took volumetric measurements of soil moisture in the nine locations previously described using a Field Scout TDR 300 soil moisture meter (Spectrum Technologies, Inc.). The instrument was inserted into the soil to a depth of 12 cm. At the same time, we measured infiltration rate at five of the nine sample locations (the center and four corner points) within each plot. At each point, we first cleared the soil surface of vegetation and thatch, then drove a 15.24 cm diameter infiltration ring into the soil. We lined the inside of the infiltration ring with plastic wrap to prevent disturbance of the soil when water was added to the ring, and poured in 444 ml (2.54 cm depth) of water. We started a timer as we removed the plastic wrap to allow the water to penetrate the soil. We stopped the timer when the water had fully infiltrated the soil. As the moisture content of the soil can affect the rate of infiltration, we repeated the process a second time in order to obtain a more accurate estimate of the infiltration rate of the soil under field‐wet conditions (; USDA, ). To ensure that infiltration was not impeded by soil saturation, soil moisture inside the infiltrometer ring was measured using a Fieldscout TDR 300 both before and after infiltration measurements (no samples were saturated).

We measured soil pH in nine locations under field‐wet conditions within each plot using a Kelway soil pH and moisture meter during March and October 2016, to see if this property varied with season. Also in October 2016, when the soil was at field moisture capacity (24 hr after rain), we measured penetration resistance with a Fieldscout SC 900 soil compaction meter (Spectrum Technologies, Inc.). Using the same nine points described earlier, we drove the soil compaction meter into the soil at approximately 2.5 cm per second and recorded the depth at which 2,068.43 kPa was reached.

### 2.4 Soil nutrient availability

We quantified the effects of elk on plant‐available nutrients in the soil using ion‐exchange probes developed by Western Ag Innovations. These probes (known as Plant Root Simulators—PRS) contain anion and cation exchange membranes that collect positively and negatively charged inorganic ions in the soil over time (the membranes are encased in plastic stakes for easy installation and recovery in the field). Ion‐exchange methods assess accumulated soil N availability over the incubation period, which can be an excellent indicator of nitrogen cycling in soils (USDA NRCS, Cherif & Loreau, 2013; Millett & Edmondson, 2015). Given that these probes measure accumulated nutrient availability over time rather than an instantaneous measure at one time, the information that they yield can be a proxy of longer‐term nitrogen availability that incorporates all pathways of nitrogen cycling, occurring in the presence of plants, accounting for plant stimulation of microbes and plant and microbial nutrient uptake.

We deployed the PRS probes to a depth of 12 cm for 8 weeks between early March and early May 2016, when plants in this Mediterranean‐type climate exhibited the greatest growth. A minimum of nine probe pairs (1 anion + 1 cation probe) were deployed evenly across each plot in the same location as other soil measurements, with extra probe pairs used in unfenced plots in order to compensate for potential losses due to elk disturbance.

After 8 weeks, the probes were retrieved, rinsed with de‐ionized water, and sent to Western Ag Innovations for analysis in order to determine the amount of ions captured in the soil. Of primary interest to us were NH_4_^+^‐N, NO_3_^‐^‐N, and PO_4_‐P, which often impact plant production and species composition in California's grasslands (see Eviner & Firestone, 2007).

### 2.5 Dung deposition

To estimate elk activity, we determined the amount of dung deposited in each of the 12 control plots of the exclosure experiment in nine surveys conducted between June 2015 and March 2016. Each survey consisted of a whole‐plot count and quantified the length and width of each dung pile. The area of an ellipse was used to estimate the area of each dung pile (in our system, dung counts, and dung area were highly correlated). As pointed out by Riginos and Grace (2008), Young, Palmer, and Gadd (2005) and others, dung counts can be used to estimate the level of activity of mammalian herbivores within habitats.

### 2.6 Statistical analyses

Our focal experiment was distributed throughout an environment that exhibited tremendous spatial heterogeneity: (a) variation in soil texture and moisture levels *within* and *among* the four known formations and (b) variation in the level of elk activity. These factors are very likely to cause spatial variation in the effects of elk on soil properties and thus were a critical component of our statistical analyses.

First, we analyzed bulk density, infiltration rate, penetration resistance, pH, nitrate, ammonium, and phosphate using linear mixed models in JMP 13 Pro (SAS Institute), with elk (present or excluded), soil formation (Kehoe 138, Kehoe 139, Sirdrak, mixed K/S soil) and their interaction as fixed effects, and sample nested within plot pair (1–12) and plot pair nested within soil formation as random effects. Response variables were transformed if they exhibited heteroscadasity or if residuals were not normally distributed.

Second, in order to examine the effects of elk, soil texture and volumetric moisture on nutrient availability, and therefore to test the hypothesis proposed by Schrama, Ciska Veen, et al. (2013), we conducted ANCOVA analyses on total inorganic nitrogen, nitrate, ammonium, and phosphate in JMP 13 Pro, with elk (present or excluded), soil texture proportion, and percent volumetric soil moisture, and their interaction as fixed effects, and sample nested within plot pair (1–12) and plot pair as random effects. Texture proportion, rather than discrete soil classification, was used in these analyses to assess the effects of texture as a continuous variable. Texture proportions and volumetric soil moisture were analyzed for correlations (Table S1), which indicated no collinearity except between two texture proportions (very coarse sand and medium sand). We further explored collinearity by putting texture and moisture variables into a model together, and checking the variance inflation factor (VIF), which is a measure of the amount of collinearity in a group of predictor variables. This showed that the texture variables were collinear (VIF > 10), but there was no collinearity between texture and soil moisture (VIF = 1.29).

To determine whether the level of elk activity affected bulk density, infiltration rate, penetration resistance, pH, nitrate, ammonium, and phosphate, we used linear regression analysis of the log response ratio (LRR) of our soil variables against total dung area (cm^2^). As elk dung only accumulated in the unfenced plots of our experiment, we used the LRR approach to condense each plot pair to a single value that could be regressed against dung area. We calculated LRR for each plot pair as follows: ln(variable mean in unfenced plot/variable mean in fenced plot). The LRR value is negative when elk has a negative effect on the variable in question (i.e., when the mean for an unfenced plot is lower than the mean for a fenced plot). Similarly, the LRR value for a plot pair is positive when the mean for an unfenced plot is higher than the exclosure mean. The further away from zero the LRR value is, the greater the magnitude of the elk effect.

Finally, an additional approach for evaluating Schrama et al.'s hypothesis was to examine whether the size of the elk effect on soil compaction contributed to the size of their effect on nutrient availability. We conducted a linear regression of the log response ratio of nutrient delivery rate as a function of the log response ratio of bulk density.

For linear regressions, data were assessed for outliers using the interquartile range method (Sullivan & La Morte, ). In this method, outliers were determined to be any data point more than 1.5 interquartile ranges (IQR) below the first quartile (25th percentile) or above the third quartile (75th percentile). Using this approach, we identified two data points as outliers and thus excluded them from our analyses, although in the interest of transparency, we included them in our graphs.

For all statistical analyses, significant effects were evaluated at *a* = 0.05 and trends (i.e., marginal effects) were evaluated at *a* = 0.10.

## 3 RESULTS

### 3.1 Soil physical properties

**FIGURE 1:**
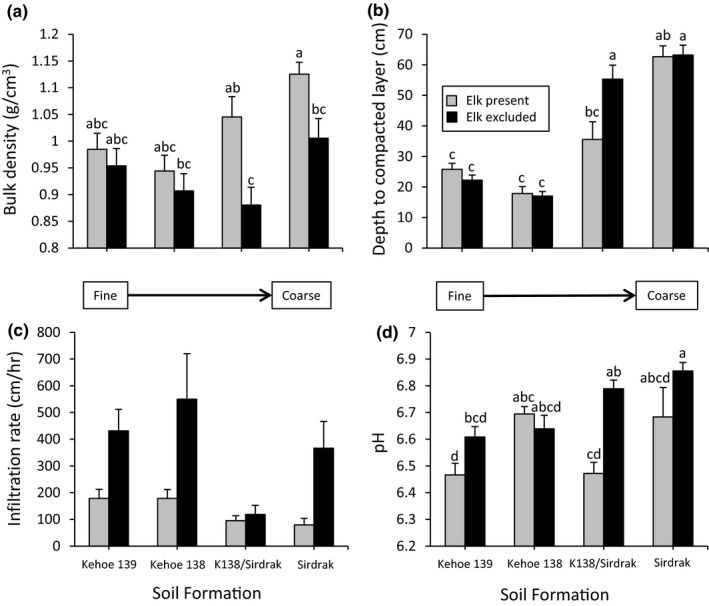
Mean (±1 *SE*) bulk density (a), compaction depth (b), infiltration rate (c), and pH (d) as a function of elk and soil formation. Letters above bars correspond to the results from Tukey multiple comparison tests

Elk increased the bulk density of soil, and this effect varied among soil formations (Table 1a). The effect was greatest on the two coarsest soils (Sirdrak sand and the mixed K/S soil) and absent on the finer soils (Kehoe 138 and Kehoe 139; Figure 1a). Elk also significantly decreased water infiltration rates, and this effect was consistent across soil formations (Table 1b). We detected a trend for elk to influence penetration resistance, and this effect varied significantly among soil formations (Table 1c), with elk causing a shallower depth of soil compaction on the mixed K/S soil (Figure 1b). Lastly, elk significantly decreased soil pH in the autumn after the first rain and this effect varied among soil formation (Table 1e), being greatest in plots with mixed K/S soil (Figure 1d).

**TABLE 1:** Results from linear mixed models evaluating the effects of tule elk (E) and soil formation (SF) on soil (a) bulk density (exponential transformed), (b) infiltration (log transformed), (c) penetration resistance, (d) pH (log transformed), (e) pH (log transformed), and (f) volumetric moisture

Response	Fixed effect	*df*	*F*	*p*	*R* ^2^(adj.)
(a) Bulk density	Elk	1, 104.2	23.70	<.0001	0.636
	SF	3, 8	0.85	.5042	
	E × SF	1, 104.1	3.49	.0183	
(b) Infiltration	Elk	1, 56.2	11.66	.0012	0.274
	SF	3, 7.5	1.95	.2057	
	E × SF	3, 56.4	1.45	.2369	
(c) Penetration resistance	Elk	1, 104	3.63	.0596	0.387
	SF	3, 8	16.76	.0008	
	E × SF	3, 104	5.63	.0013	
(d) pH (3/16)	Elk	1, 92.6	1.38	.2432	0.459
	SF	3, 6.6	19.14	.0012	
	E × SF	3, 92.5	1.23	.3053	
(e) pH (10/16)	Elk	1, 104	12.65	.0006	−0.16
	SF	3, 8	4.38	.0421	
	E × SF	2, 104	4.01	.0095	
(f) Moisture (3/16)	Elk	1, 63.4	0.46	.5024	0.542
	SF	3, 7.8	11.68	.0029	
	E × SF	3, 63.4	1.43	.2423	

### 3.2 Soil nutrient availability

**FIGURE 2:**
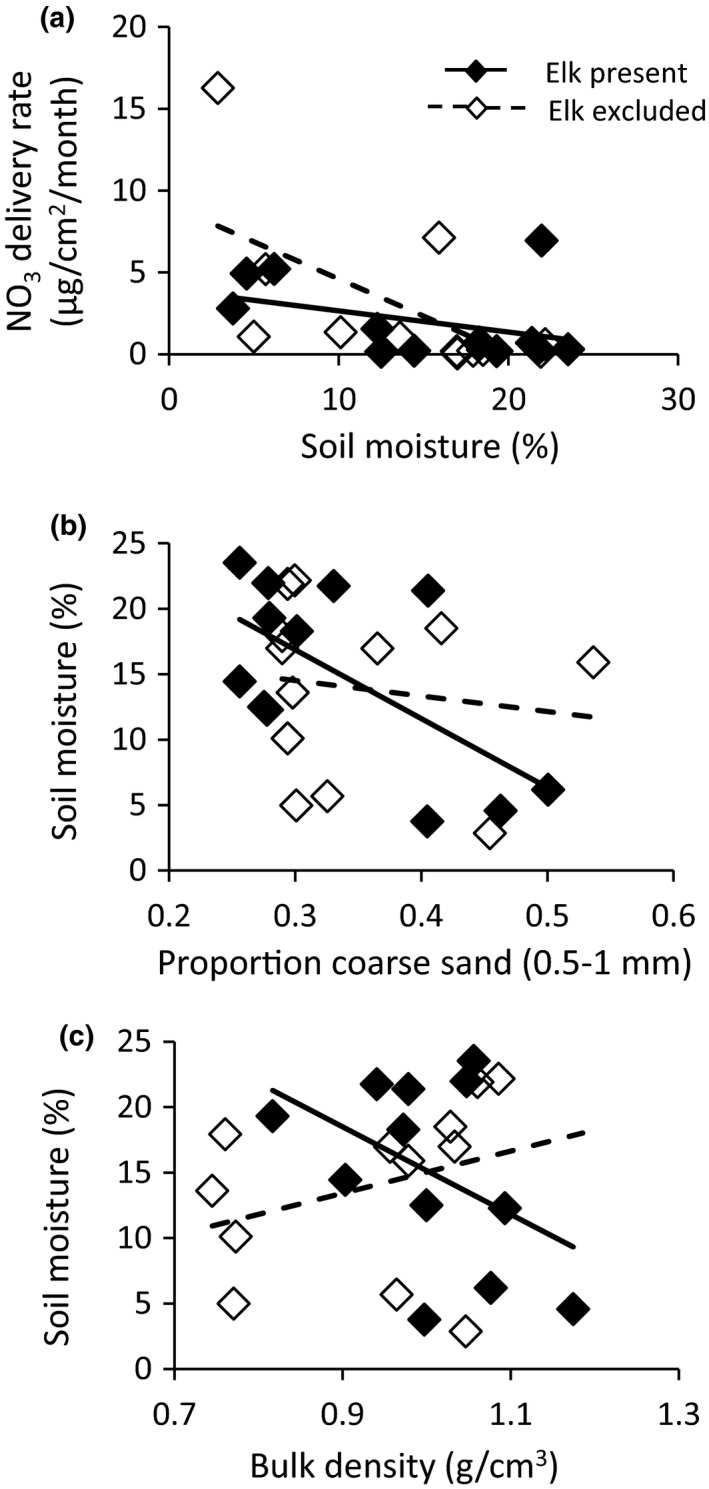
The effect of elk and soil moisture on nitrate delivery rate (a), the effect of elk and proportion of coarse sand (0.5–1 mm) on % soil moisture (b), and the effect of elk and bulk density on % soil moisture (c)

**FIGURE 3:**
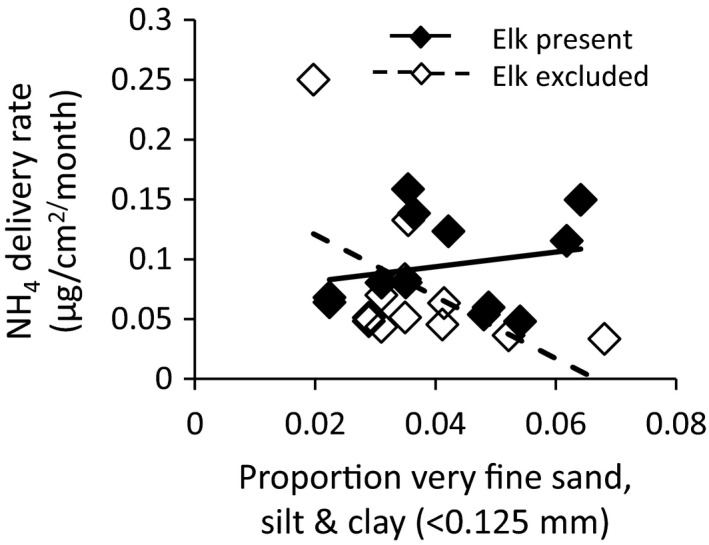
The effect of elk and proportion of very fine sand/silt/clay on ammonium delivery rate

When soil formation was treated as a categorical variable in our linear mixed models, we did not detect an effect of elk on nitrate, ammonium, and phosphate availability across all soil formations, nor was there a significant interaction between elk and soil formation (Table 2). However, when the physical differences among the four soil formations were treated as continuous variables, we detected significant effects of elk on nitrogen availability. Elk influenced both soil moisture and the effects of soil moisture on nitrogen availability (Table 3e; Table 4b). They decreased nitrate availability in drier soils (<16% moisture content), and increased nitrate availability at higher levels of soil moisture (>16% soil moisture; Table 4b; Figure 2a). With increasing proportion of coarse sand, elk decreased soil moisture content (Table 3e; Figure 2b), possibly through their positive effects on soil bulk density (Table 1a; Figure 2c), which were proportionally larger on coarser soils (Table 1a; Figure 1a). The effects of elk on ammonium availability varied with soil texture (Table 5c), with effects being greatest on soils with higher proportions of very fine particles (Figure 3).

**TABLE 2:** Results from linear mixed models evaluating the effects of tule elk and soil formation (SF) on soil nutrient availability 2016 (a) Total inorganic N (ln transformed), (b) NO3‐N (ln transformed), (c) NH4 (log transformed), and (d) Phosphate (log transformed)

Response	Fixed effect	*df*	*F*	*p*	*R* ^2^(adj.)
(a) Total inorganic N	Elk	1, 39.6	0.11	.7466	0.598
	SF	3, 9.3	16.42	.0005	
	E × SF	3, 39.2	2.03	.1253	
(b) NO3	Elk	1, 37.6	0.03	.8581	0.285
	SF	3, 8.4	9.11	.0051	
	E × SF	3, 37.7	1.29	.2925	
(c) NH4	Elk	1, 42.3	2.78	.1028	0.298
	SF	3, 7.6	15.38	.0013	
	E × SF	3, 42	1.11	.3568	
(d) Phosphate	Elk	1, 44.1	0.26	.6141	
	SF	3, 8.3	3.08	.0885	
	E × SF	1, 43.8	0.46	.7108	

**TABLE 3:** Results from ANCOVA analyses evaluating the effects of tule elk and proportion of coarse sand (0.5–1 mm) on 2016 soil nutrient availability (a) Total inorganic N (ln transformed), (b) NO3‐N (ln transformed), (c) NH4 (log transformed), and (d) Phosphate (log transformed)

Response	Fixed effect	*df*	*F*	*p*
(a) Total inorganic N	Elk	1, 37.2	0.00	.9757
	Texture	1, 26.8	0.31	.5806
	E × T	1, 42	0.01	.9804
(b) NO3	Elk	1, 37.2	0.08	.786
	Texture	1, 14.4	1.60	.2266
	E × T	1, 45.3	0.16	.6903
(c) NH4	Elk	1, 41.9	1.54	.2219
	Texture	1, 16.6	0.06	.8115
	E × T	1, 49	0.16	.6903
(d) Phosphate	Elk	1, 45.9	0.79	.379
	Texture	1, 35.4	1.03	.3164
	E × T	1, 49.2	0.00	.988
(e) Volumetric moisture	Elk	1, 63.3	1.72	.1943
	Texture	1, 74.2	1.58	.2131
	E × T	1, 66.6	6.76	.0115

**TABLE 4:** Results from ANCOVA analyses evaluating the effects of tule elk and percent soil moisture on 2016 soil nutrient availability (a) Total inorganic N (ln transformed), (b) NO3‐N (ln transformed), (c) NH4 (log transformed), and (d) Phosphate (log transformed)

Response	Fixed effect	*df*	*F*	*p*
(a) Total inorganic N	Elk	1, 43.1	0.21	.6479
	Moisture	1, 20.2	14.47	.0011
	E × M	1, 47	4.51	.0389
(b) NO3	Elk	1, 41.1	0.52	.477
	Moisture	1, 14.7	17.34	.0009
	E × M	1, 43.4	3.94	.0535
(c) NH4	Elk	1, 44.3	2.01	.1632
	Moisture	1, 14.2	2.91	.1099
	E × M	1, 51	0.77	.3852
(d) Phosphate	Elk	1, 46	0.78	.3821
	Moisture	1, 28.1	0.51	.4828
	E × M	1, 49	0.03	.8591

**TABLE 5:** Results from ANCOVA analyses evaluating the effects of tule elk and proportion of very fine sand/silt/clay (<0.125 mm) on 2016 soil nutrient availability (a) Total inorganic N (ln transformed), (b) NO3‐N (ln transformed), (c) NH4 (log transformed), and (d) Phosphate (log transformed)

Response	Fixed effect	*df*	*F*	*p*
(a) Total inorganic N	Elk	1, 45.8	0.15	.7016
	Texture	1, 33.6	0.79	.3809
	E × T	1, 44.8	1.10	.3005
(b) NO3	Elk	1, 42.3	0.00	.9775
	Texture	1, 20.7	0.64	.4314
	E × T	1, 41.4	0.43	.514
(c) NH4	Elk	1, 45.8	3.54	.0663
	Texture	1, 22.9	1.53	.2290
	E × T	1, 45.9	7.35	.0094
(d) Phosphate	Elk	1, 48.9	0.41	.5237
	Texture	1, 32.6	0.07	.7887
	E × T	1, 47.8	1.15	.2893
(e) Volumetric moisture	Elk	1, 69.7	2.63	.1095
	Texture	1, 70.2	1.53	.2200
	E × T	1, 64.5	1.49	.2266

**FIGURE 4:**
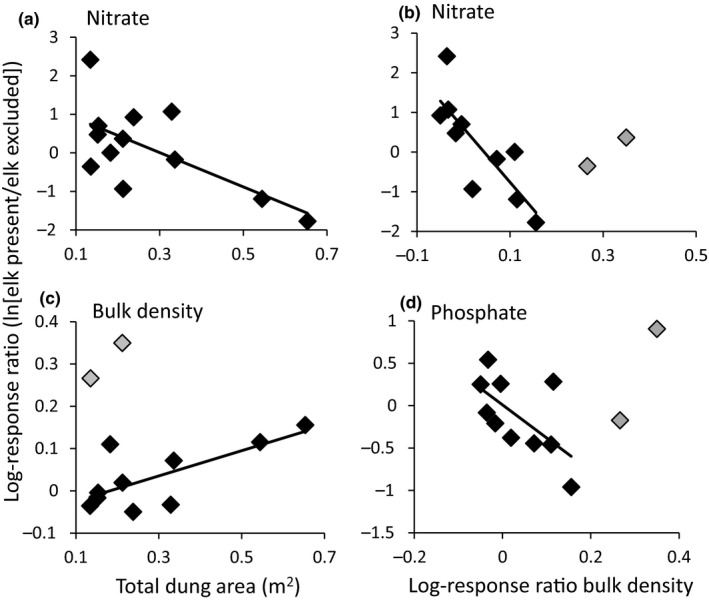
The log response ratio of nitrate as a function of total dung area (a); the log response ratio of bulk density as a function of total dung area (data identified as outliers via interquartile range method in gray; b); log response ratio of nitrate as a function of the log response ratio of bulk density (outliers in gray; c); and the log response ratio of phosphate as a function of the log response ratio of bulk density (outliers in gray; d)

With increasing elk activity (as measured by total dung area), bulk density increased (Table 6a; Figure 4a,c). Nitrate availability decreased with increased elk activity (Table 6c) and elk‐induced increases in bulk density strongly correlated with their decrease in the availability of nitrate (Table 7b; Figure 4b). There was not a significant relationship between ammonium availability and elk activity level (Table 6d) or bulk density (Table 7c).

**TABLE 6:** Results from linear regressions evaluating the effects of total dung area (cm^2^) on (a) Log response ratio (LRR) Total inorganic N, (b) LRR NO3, (c) LRR NH4, and (d) LRR Phosphate

Response	Fixed effect	*df*	*F*	*p*	*R* ^2^
(a) LRR Bulk density	Tot. dung	1, 9	8.43	.0198	0.51
(b) LRR total inorganic N	Tot. dung	1, 10	8.60	.015	0.462
(c) LRR NO3	Tot. dung	1, 10	7.70	.0196	0.435
(d) LRR NH4	Tot. dung	1, 10	0.00	.9775	8.3e−5
(e) LRR Phosphate	Tot. dung	1, 10	0.80	.3913	0.074

**TABLE 7:** Results from linear regressions evaluating the effects of log response ratio of bulk density (LRR BD) with outliers removed on (a) Log response ratio (LRR) Total inorganic N, (b) LRR NO3, (c) LRR NH4, and (d) LRR Phosphate

Response	Fixed effect	*df*	*F*	*p*	*R* ^2^
(a) LRR total inorganic N	LRR BD	1, 10	14.92	.0048	0.607
(b) LRR NO3	LRR BD	1, 10	15.98	.004	0.625
(c) LRR NH4	LRR BD	1, 10	1.17	.3103	0.018
(d) LRR Phosphate	LRR BD	1, 10	5.27	.0508	0.322

Elk did not have a significant effect on phosphate availability, nor was there a significant interaction between elk and soil formation (Table 2d). There was a trend for phosphate availability to vary with soil formation (Table 2d), with levels being higher in the Kehoe 138 soil formation than all other soils. Elk did not interact with soil moisture or texture to affect this nutrient, nor did the level of elk activity have a significant effect on phosphate (Table 4d; Table 6e). However, when we looked at the relationship between bulk density and phosphate, we found that as elk increased bulk density they decreased the availability of phosphate (Table 7d; Figure 4d).

## 4 DISCUSSION

Understanding the drivers of variability in the effects of herbivores on ecosystems has been a daunting challenge, and a key frontier in managing and predicting ecosystem responses to herbivory (Hobbs 1996; Andriuzzi & Wall, 2017; Forbes et al., 2019; Ritchie et al., 1998; Schrama, Ciska Veen, et al., 2013; Sitters & Olde Venterink, 2015; Wardle et al., 2004). Using an 18‐year‐old exclosure experiment stratified across distinct soil formations differing in texture, moisture content, plant biomass, and herbivore use, our study addressed the complex interactions of factors that drive the effects of herbivores on ecosystems. A reintroduced, native herbivore—tule elk—altered many soil properties, and the effects on any given soil measure had a wide range of variability. Variation in the effects of tule elk on soil properties was driven by heterogeneity in soil conditions, but only some of the soil responses to herbivory (e.g., bulk density) varied strongly with soil formation, when considered as a categorical variable. Other soil responses to herbivory (e.g., nitrogen availability, soil moisture) were only detectable when we analyzed soil differences as continuous rather than categorical variables. The importance of moving beyond simple soil type × herbivory interactions was also highlighted by the fact that many controlling factors of ecosystem processes varied independently from one another. The differences across soil types in plant biomass, herbivore activity, and soil moisture were not parallel to one another, thus further stressing the need to move beyond broad soil categories to understand the context‐dependent effects of herbivores on ecosystems.

As seen in studies with other herbivores (Castellano & Malone, 2007; Heckel, Bourg, McShea, & Kalisz, 2010; Gass & Binkley, 2011; Daryanto et al., 2013), our study showed that tule elk increased bulk density, decreased infiltration rate, and led to the formation of a more compact soil layer, with these effects amplifying at higher levels of elk activity. It is generally assumed that finer soils are more compactible due to the fact that they tend to store water in their pore spaces, and more force is required to compact a dry soil than a moist or wet soil (Lull, ; Reynolds & Packer, ; Van Haveren, 1983). Elk only affected the compacted layer depth in the mixed fine‐coarse soil, and not in the coarse soil, which is in keeping with Schrama, Heijning, et al. (2013) findings that bulk density effects can occur in both sandy and clay soils, but that the ecological consequences of this change in bulk density are less pronounced in sandy soils. However, contrary to our expectations, we found that elk had the strongest effect on bulk density in coarser soils (Sirdrak sand and mixed K/S soil). We hypothesize that this is in part due to the fact that one of the two plots in our study system where elk spend the most time (based on dung counts and personal observation) is on top of Sirdrak sand (Figure S1). The other plot where elk spend the most time is on top of Kehoe 139 soil. According to Vallentine (1990), Ampe, Langohr, and Ngugi () and others, soils covered by dense sod (like the Kehoe 139) are less susceptible to damage and other changes from trampling, due the concentrated root network in the soil acting as a buffer against root compaction. The Sirdrak sand is not covered by dense sod, rather, this loose, structureless sand features many patches of bare ground between plant cover, and therefore may be more susceptible to compaction than the other soils in the system.

In our study, elk decreased soil pH in most soil types, as seen in other studies (Binkley et al., 2003; Lucas et al., 2013). Elk likely affected pH via numerous pathways. Preferential feeding by elk may reduce the amount of alkaline or neutral leaf litter reaching soil, and since acidic plants are less palatable (Rhodes, Anderson, St, & Clair., 2017), more acidic leaf litter may be accumulating in the soil. Feeding by elk can induce plants to release root exudates that decrease soil pH (Hinsinger, Plassard, Tang, & Benoît, 2003). Also, mineralogy of the underlying parent rock may create chemical conditions that interact with elk metabolic wastes, resulting in different pH conditions in different soils (Haynes & Williams, 1992; Irmak, Surucu, & Aydogdu, 2007; Neff, Reynolds, Sanford, Fernandez, & Lamothe, 2006). In addition, elk may alter the pH of the soil through deposition of their metabolic wastes. Urea hydrolyzes slowly on dry soil, but after rainfall, it rapidly hydrolyzes to ammonium and cyanate, resulting in an initial rise in soil pH (Black, Sherlock, & Smith, 1987). Volatilization of ammonia from the soil surface can then result in a decrease in soil pH, because the conversion of ammonium to ammonia results in a release of H+ ions in the soil (Bolan, Hedley, & White, 1991). This decrease in pH can, in turn, indirectly affect plant growth through influencing the availability of certain nutrients, such as iron and manganese (Killham, 1994).

The only effect of elk on phosphate availability that we observed was indirect, mediated by their effect on bulk density. Increased bulk density decreases pore space in the soil and can lead to encapsulation of soil moisture, which might limit phosphate diffusion and uptake by plants. The effects of elk on bulk density might also have resulted in a change in the soil microbial community. Previous studies have shown that an increase in bulk density can result in a reduction in microbial biomass phosphate as well as reduced soil enzymatic activity, including the activity of phosphatase (Dick, Myrold, & Kerle, 1988; Pupin, da Silva Freddi, & Nahas, 2008; Tan, Chang, & Kabzems, 2008). Both reductions in microbial biomass and phosphatase can lead to less available phosphate in the soil.

Our study offered the opportunity to examine aspects of different frameworks for predicting variation in the effects of herbivores on nitrogen mineralization. The framework posited by Wardle, Hobbs, Ritchie and others proposes that herbivores will affect nutrient cycling through their effects on the plant community. In our study system, Johnson and Cushman (2007) found that elk decreased shrub cover (lower quality, high C:N plants) and promoted growth of non‐woody species (higher quality, lower C:N plants). Based on this plant‐quality framework, we would expect that by increasing plants with higher quality leaf litter, elk would increase nitrogen mineralization and availability in our system. However, the effects of elk were variable, with both increases and decreases in nitrogen availability being observed, depending on soil texture and moisture. Across our entire study, increasing elk activity was correlated with a decrease in nitrate availability. Our findings echo many other studies (Bakker et al., 2004; Millett & Edmondson, 2015; Schrama, Heijning, et al., 2013; Sitters et al., 2017; Stark & Grellmann, 2002; Vaieretti et al., 2013), indicating that the plant‐quality framework does not fully explain the variable effects of herbivores on nitrogen cycling.

Our nitrate results are in line with the modified framework proposed by Schrama, Ciska Veen, et al. (2013), in that elk had a negative influence on nitrate in coarse, dry soils, but follow the expectations of the plant‐quality framework at intermediate levels of moisture. It is important to reiterate that the moisture‐dependent effects of herbivory on nitrate were only evident by analyzing the effects of elk along a moisture gradient rather than soil formation as a categorical variable. This is likely because, in addition to the texture differences across soil formations, within each soil formation, there was high variation in soil moisture content and the proportions of each texture category (very coarse, coarse, medium, fine, and very fine). For instance, based on mean soil moisture, the Kehoe 138 and 139 formations would fit into the intermediate moisture category as defined by the framework of Schrama, Ciska Veen, et al. (2013). But some individual cores within these soil types would have been classified as dry. Similarly, both the Sirdrak sand and the mixed K/S soil would be classified as coarse, dry soils based on mean soil moisture, but also possessed areas of intermediate moisture content. Therefore, any effects of elk on nitrate could have been masked by this variability in soil moisture content when only mean levels of soil moisture were considered for each formation.

The framework proposed by Schrama, Ciska Veen, et al. (2013) also predicts that herbivores should negatively affect nitrate availability through their positive effect on soil bulk density. Our results lend support for this idea. When we plotted LRR of nitrate against LRR bulk density, we found that the more positive the elk effect was on bulk density, the more negative their effect was on nitrate availability in 10 of the 12 plot pairs in our system (Figure 4c). The two outlier plot pairs that did not follow this trend also did not follow the trend for bulk density increasing with increasing level of elk activity (Figure 4b). This may be due to these two plot pairs being underlain by extremely heterogeneous geology (Clark, Brabb, Greene, & Ross, 1984; Stoffer, ), and the resulting soils may be more compactible. In summary, as is evident by the number of frameworks developed to explain the context‐dependent effects of herbivores on soils, understanding the site‐specific effects of herbivores requires consideration of the interactions across multiple driving variables. As predicted by the framework put forward by Schrama, Ciska Veen, et al. (2013), in soils of intermediate moisture content, elk increased nitrate availability and favored plant species with higher quality leaf litter, consistent with the predictions of the plant‐quality framework. These finer soils also had no effects of elk on bulk density or depth to compacted layer.

In the coarser, drier soils, where elk significantly increased bulk density, elk decreased soil moisture and decreased nitrate availability. These negative effects are predicted by Schrama, Ciska Veen, et al. (2013) in these dry soils, where the effects of large herbivores on increasing bulk density would override their effects on improved litter quality. Numerous studies have shown that compaction by herbivores decreases pore spaces, leading to lower infiltration rates and lower water storage (Abdel‐Magid et al., 1987; Daryanto et al., 2013; Gass & Binkley, 2011; Steffens et al., 2008; Thrash, 1997). We hypothesize that this is how elk modified the effect of soil moisture on nitrate availability (Figure 2a). Additionally, in more compacted soils, low air‐filled porosity can result in denitrification and loss of inorganic nitrogen via gaseous emissions (Gregorich, McLaughlin, Lapen, Ma, & Rochette, 2014; Torbert & Wood, 1992).

Elk effects on phosphate availability were mediated by their positive effects on bulk density, which reduced available phosphate (Figure 4d). This could be due to decreased pore space in the soil, which could limit phosphate diffusion and uptake by plants. The effects of elk on phosphate were only evident through this relationship—there was not a significant direct effect of elk when measured as a categorical variable or by elk activity as a continuous variable.

In conclusion, our study has demonstrated that both large herbivores and the physical properties of soils can be important drivers of ecosystem‐level processes and can interact to produce context‐dependent effects of herbivores on these processes. These context‐dependent effects are predictable by considering gradients of herbivore activity, soil texture, and moisture levels. Our approach provides a guideline for future research focused on how large herbivores influence soil properties and process across heterogeneous conditions, and can be expanded upon by looking across more diverse sites where stress gradients can moderate the effects of herbivores (Andriuzzi & Wall, 2017). Improved ability to make such predictions will help inform land managers about areas of the landscape that are more vulnerable to degradation from livestock and native herbivores, and help to set grazing regimes that are sustainable for multiple goals—a key challenge in ecosystem management with herbivores (Briske, Derner, Milchunas, & Tate, ; Teague & Barnes, 1997). This can also help forecast possible outcomes of reintroducing large herbivores, with a view toward ensuring long‐term success of such reintroduction efforts.

## CONFLICT OF INTEREST

All three authors certify that they do not have any conflict of interest to disclose.

## AUTHOR CONTRIBUTIONS

**Vanessa J. Dodge:** Conceptualization (supporting); formal analysis (lead); investigation (equal); methodology (lead); visualization (equal); writing – original draft (equal); writing – review & editing (equal). **Valerie T. Eviner:** Investigation (equal); visualization (equal); writing – review & editing (equal). **J. Hall Cushman:** Conceptualization (lead); formal analysis (equal); funding acquisition (lead); investigation (equal); methodology (equal); project administration (lead); supervision (lead); writing – review & editing (equal).

## Supporting information

Fig S1

Table S1

Fig S1‐cap

## Data Availability

Data used in this paper are published on Dryad—https://doi.org/10.5061/dryad.bvq83bk6k.

## References

[ece36743-bib-0099] Abbas, F. , Merlet, J. , Morellet, N. , Verheyden, H. , Hewison, A.J.M. , Cargnelutti, B. , & Aulagnier, S. (2012). Roe deer may markedly alter forest nitrogen and phosphorus budgets across Europe. Oikos, 121(8), 1271–1278.

[ece36743-bib-0001] Abdel‐Magid, A. H. , Schuman, G. E. , & Hart, R. H. (1987). Soil bulk density and water infiltration as affected by grazing systems. Journal of Range Management, 40, 307–309. 10.2307/3898725

[ece36743-bib-0003] Andriuzzi, W. S. , & Wall, D. H. (2017). Responses of belowground communities to large herbivores: Meta‐analysis reveals biome‐dependent patterns and critical research gaps. Global Change Biology, 23, 3857–3868. 28245090 10.1111/gcb.13675

[ece36743-bib-0004] Augustine, D. J. , & McNaughton, S. J. (2006). Interactive effects of ungulate herbivores, soil fertility, and variable rainfall on ecosystem processes in a semi‐arid savanna. Ecosystems, 9, 1242–1256. 10.1007/s10021-005-0020-y

[ece36743-bib-0005] Bailey, D. W. , Gross, J. E. , Laca, E. A. , Rittenhouse, L. R. , Coughenour, M. B. , Swift, D. M. , & Sims, P. L. (1996). Mechanisms that result in large herbivore grazing distribution patterns. Journal of Range Management, 49, 386–400. 10.2307/4002919

[ece36743-bib-0006] Bakker, E. S. , Knops, J. M. H. , Milchunas, D. G. , Ritchie, M. E. , & Olff, H. (2009). Cross‐site comparison of herbivore impact on nitrogen availability in grasslands: The role of plant nitrogen concentration. Oikos, 118, 1613–1622. 10.1111/j.1600-0706.2009.17199.x

[ece36743-bib-0007] Bakker, E. S. , Olff, H. , Boekhoff, M. , Gleichman, J. M. , & Berendse, F. (2004). Impact of herbivores on nitrogen cycling: Contrasting effects of small and large species. Oecologia, 138, 91–101. 10.1007/s00442-003-1402-5 14566555

[ece36743-bib-0008] Bardgett, R. D. , & Wardle, D. A. (2003). Herbivore‐mediated linkages between aboveground and belowground communities. Ecology, 84, 2258–2268. 10.1890/02-0274

[ece36743-bib-0009] Bardgett, R. D. , Wardle, D. A. , & Yeates, G. W. (1998). Linking above‐ground and below‐ground interactions: How plant responses to foliar herbivory influence soil organisms. Soil Biology and Biochemistry, 30, 1867–1878. 10.1016/S0038-0717(98)00069-8

[ece36743-bib-0010] Binkley, D. , Singer, F. , Kaye, M. , & Rochelle, R. (2003). Influence of elk grazing on soil properties in Rocky Mountain National Park. Forest Ecology and Management, 185, 239–247. 10.1016/S0378-1127(03)00162-2

[ece36743-bib-0011] Black, A. S. , Sherlock, R. R. , & Smith, N. P. (1987). Effect of timing of simulated rainfall on ammonia volatilization from urea, applied to soil of varying moisture content. Journal of Soil Science, 38, 679–687. 10.1111/j.1365-2389.1987.tb02165.x

[ece36743-bib-0012] Bolan, N. S. , Hedley, M. J. , & White, R. E. (1991). Processes of soil acidification during nitrogen cycling with emphasis on legume‐based pastures. Plant and Soil, 134, 53–63. 10.1007/BF00010717

[ece36743-bib-0014] Buckeridge, K. M. , & Jefferies, R. L. (2007). Vegetation loss alters soil nitrogen dynamics in an arctic salt marsh. Journal of Ecology, 95, 283–293. 10.1111/j.1365-2745.2007.01214.x

[ece36743-bib-0015] Castellano, M. J. , & Malone, T. J. (2007). Livestock, soil compaction and water infiltration rate: Evaluating a potential desertification recovery mechanism. Journal of Arid Environments, 71, 97–108. 10.1016/j.jaridenv.2007.03.009

[ece36743-bib-0016] Cecil, E. , Spasojevic, M. J. , & Cushman, J. H. (2019). Cascading effects of mammalian herbivores on ground‐dwelling arthropods: Variable responses across arthropod groups, habitats and seasons. Journal of Animal Ecology, 88, 1319–1331. 31135962 10.1111/1365-2656.13033

[ece36743-bib-0017] Chaneton, E. J. , & Lavado, R. S. (1996). Soil nutrients and salinity after long‐term grazing exclusion in a flooding pampa grassland. Journal of Range Management, 49, 182–187. 10.2307/4002692

[ece36743-bib-0018] Cherif, M. , & Loreau, M. (2013). Plant–herbivore–decomposer stoichiometric mismatches and nutrient cycling in ecosystems. Proceedings of the Royal Society B: Biological Sciences, 280(1754), 20122453 10.1098/rspb.2012.2453 PMC3574320 23303537

[ece36743-bib-0019] Clark, J. C. , Brabb, E. E. , Greene, H. G. , & Ross, D. C. (1984). Geology of Point Reyes Peninsula and implications for San Gregorio fault history, tectonics and sedimentation along the California margin. Society of Economic Paleontologists and Mineralogists, Pacific Section, 38, 67–86.

[ece36743-bib-0020] Daryanto, S. , Eldridge, D. J. , & Wang, L. (2013). Spatial patterns of infiltration vary with disturbance in a shrub‐encroached woodland. Geomorphology, 194, 57–64. 10.1016/j.geomorph.2013.04.012

[ece36743-bib-0021] Dick, R. P. , Myrold, D. D. , & Kerle, E. A. (1988). Microbial biomass and soil enzyme activities in compacted and rehabilitated skid trail soils. Soil Science Society of America Journal, 52, 512–516. 10.2136/sssaj1988.03615995005200020038x

[ece36743-bib-0022] Dodge, V. J. (2017). Effects of a reintroduced herbivore on ecosystem‐level processes: An integrated approach for understanding variable outcomes across heterogeneous landscapes. master's thesis. Sonoma State University.

[ece36743-bib-0023] Ellis, T. D. , & Cushman, J. H. (2018). Indirect effects of a large mammalian herbivore on small mammal populations: Context‐dependent variation across habitat types, mammal species and seasons. Ecology & Evolution, 8, 12115–12125. 10.1002/ece3.4670 30598804 PMC6303759

[ece36743-bib-0024] Ender, C. , Christian, C. , & Cushman, J. H. (2017). Native herbivores and environmental heterogeneity as mediators of an exotic grass invasion. Ecology and Evolution, 7, 1561–1571. 10.1002/ece3.2727 28261465 PMC5330880

[ece36743-bib-0025] Eviner, V. T. , & Firestone, M. K. (2007). Mechanisms determining patterns of nutrient dynamics In Stromberg M., Corbin J., & D'Antonio C. (Eds.), California grasslands: Ecology and management (pp. 94–106). Berkeley, CA: University of California Press.

[ece36743-bib-0026] Forbes, E. S. , Cushman, J. H. , Burkepile, D. E. , Young, T. P. , Klope, M. , & Young, H. S. (2019). Synthesizing the effects of large, wild herbivore exclusion on ecosystem function. Functional Ecology, 33, 1597–1610. 10.1111/1365-2435.13376

[ece36743-bib-0027] Ford, M. A. , & Grace, J. B. (1998). Effects of vertebrate herbivores on soil processes, plant biomass, litter accumulation and soil elevation changes in a coastal marsh. Journal of Ecology, 86, 974–982. 10.1046/j.1365-2745.1998.00314.x

[ece36743-bib-0028] Frank, D. A. , Gehring, C. A. , Machut, L. , & Phillips, M. (2003). Soil community composition and the regulation of grazed temperate grassland. Oecologia, 137, 603–609. 10.1007/s00442-003-1385-2 14513350

[ece36743-bib-0029] Frank, D. A. , & Groffman, P. M. (1998). Ungulate vs. landscape control of soil C and N processes in grasslands of Yellowstone National Park. Ecology, 79, 2229–2241.

[ece36743-bib-0030] Frank, D. A. , Groffman, P. M. , Evans, R. D. , & Tracy, B. F. (2000). Ungulate stimulation of nitrogen cycling and retention in Yellowstone Park Grasslands. Oecologia, 123, 116–121. 10.1007/s004420050996 28308736

[ece36743-bib-0031] Frank, D. A. , Inouye, R. S. , Huntly, N. , Minshall, G. W. , & Anderson, J. E. (1994). The biogeochemistry of a north‐temperate grassland with native ungulates: Nitrogen dynamics in Yellowstone National Park. Biogeochemistry, 26, 163–188. 10.1007/BF00002905

[ece36743-bib-0032] Frank, D. A. , & McNaughton, S. J. (1993). Evidence for the promotion of aboveground grassland production by native large herbivores in Yellowstone National Park. Oecologia, 96, 157–161. 10.1007/BF00317727 28313410

[ece36743-bib-0033] Frost, C. J. , & Hunter, M. D. (2007). Recycling of nitrogen in herbivore feces: Plant recovery, herbivore assimilation, soil retention, and leaching losses. Oecologia, 151, 42–53. 10.1007/s00442-006-0579-9 17089141

[ece36743-bib-0034] Gass, T. M. , & Binkley, D. (2011). Soil nutrient losses in an altered ecosystem are associated with native ungulate grazing. Journal of Applied Ecology, 48, 952–960. 10.1111/j.1365-2664.2011.01996.x

[ece36743-bib-0035] Gogan, P. J. , & Barrett, R. H. (1995). Elk and deer diets in a coastal prairie‐scrub mosaic, California. Journal of Range Management, 48, 327–335. 10.2307/4002485

[ece36743-bib-0036] Gregorich, E. G. , McLaughlin, N. B. , Lapen, D. R. , Ma, B. L. , & Rochette, P. (2014). Soil compaction, both an environmental and agronomic culprit: Increased nitrous oxide emissions and reduced plant nitrogen uptake. Soil Science Society of America Journal, 6, 1913–1923. 10.2136/sssaj2014.03.0117

[ece36743-bib-0037] Hatton, J. C. , & Smart, N. O. E. (1984). The effect of long‐term exclusion of large herbivores on soil nutrient status in Murchison Falls National Park, Uganda. African Journal of Ecology, 22, 23–30. 10.1111/j.1365-2028.1984.tb00670.x

[ece36743-bib-0038] Haynes, R. J. , & Williams, P. H. (1992). Changes in soil solution composition and pH in urine‐affected areas of pasture. European Journal of Soil Science, 43, 323–334. 10.1111/j.1365-2389.1992.tb00140.x

[ece36743-bib-0039] Heckel, C. D. , Bourg, N. A. , McShea, W. J. , & Kalisz, S. (2010). Nonconsumptive effects of a generalist ungulate herbivore drive decline of unpalatable forest herbs. Ecology, 91, 319–326. 10.1890/09-0628.1 20391995

[ece36743-bib-0040] Hempson, G. P. , Archibald, S. , Bond, W. J. , Ellis, R. P. , Grant, C. C. , Krurger, F. J. , … Vickers, K. J. (2014). Ecology of grazing lawns in Africa. Biological Reviews, 9, 979–994. 10.1111/brv.12145 25231416

[ece36743-bib-0041] Hinsinger, P. , Plassard, C. , Tang, C. , & Benoît, J. (2003). Origins of root‐mediated pH changes in the rhizosphere and their responses to environmental constraints: A review. Plant and Soil, 248, 43–59. 10.1023/A:1022371130939

[ece36743-bib-0042] Hobbs, T. , & Thompson, N. (1996). Modification of ecosystems by ungulates. The Journal of Wildlife Management, 60, 695–713. 10.2307/3802368

[ece36743-bib-0043] Homburger, H. , Luscher, A. , Scherer‐Lorenzen, M. , & Schneider, M. K. (2015). Patterns of livestock activity on heterogeneous subalpine pastures reveal distinct responses to spatial autocorrelation, environment and management. Movement Ecology, 3, 35 10.1186/s40462-015-0053-6 26457186 PMC4598957

[ece36743-bib-0044] Irmak, S. , Surucu, A. K. , & Aydogdu, A. H. (2007). Effects of different parent material on the mineral characteristics of soils in the arid region of Turkey. Pakistan Journal of Biological Sciences, 10, 528–536. 10.3923/pjbs.2007.528.536 19069531

[ece36743-bib-0045] Johnson, B. E. , & Cushman, J. H. (2007). Influence of a large herbivore reintroduction on plant invasions and community composition in a California grassland. Conservation Biology, 21, 515–526. 10.1111/j.1523-1739.2006.00610.x 17391201

[ece36743-bib-0046] Kashiwagi, J. H. (1985). Soil survey of Marin County, California. Soil Conservation Service.

[ece36743-bib-0047] Killham, K. (1994). Soil ecology, Cambridge, UK: Cambridge University Press.

[ece36743-bib-0048] Kleinhesselink, A. R. , Magnoli, S. M. , & Cushman, J. H. (2014). Shrubs as ecosystem engineers across an environmental gradient: Effects on species richness and exotic plant invasion. Oecologia, 175, 1277–1290. 10.1007/s00442-014-2972-0 24871135

[ece36743-bib-0049] Lavado, R. S. , & Alconada, M. (1994). Soil properties behavior on grazed and ungrazed plots of a grassland sodic soil. Soil Technology, 7, 75–81. 10.1016/0933-3630(94)90008-6

[ece36743-bib-0050] Lucas, R. W. , Salguero‐Gómez, R. , Cobb, D. B. , Waring, B. G. , Anderson, F. , McShea, W. J. , & Casper, B. B. (2013). White‐tailed deer (*Odocoileus virginianus*) positively affect the growth of mature northern red oak (*Quercus rubra*) trees. Ecosphere, 4, 1–15.

[ece36743-bib-0052] McCullough, D. R. (1969). The Tule elk: Its history, behavior, and ecology, Berkeley, CA: University of California Press.

[ece36743-bib-0053] McNaughton, S. J. , Banyikwa, F. F. , & McNaughton, M. M. (1997). Promotion of the cycling of diet‐enhancing nutrients by African grazers. Science, 278, 1798–1800. 10.1126/science.278.5344.1798 9388182

[ece36743-bib-0054] McNaughton, S. J. , Ruess, R. , & Seagle, S. (1988). Large mammals and process dynamics in African ecosystems. BioScience, 38, 794–800. 10.2307/1310789

[ece36743-bib-0056] McNeil, S. G. , & Cushman, J. H. (2005). Indirect effects of deer herbivory on local nitrogen availability in a coastal dune ecosystem. Oikos, 110, 124–132. 10.1111/j.0030-1299.2005.13686.x

[ece36743-bib-0057] McSherry, M. E. , & Ritchie, M. E. (2013). Effects of grazing on grassland soil carbon: A global review. Global Change Biology, 19, 1347–1357. 10.1111/gcb.12144 23504715

[ece36743-bib-0058] Millett, J. , & Edmondson, S. (2015). The impact of 36 years of grazing management on soil nitrogen (N) supply rate and *Salix repens* N status and internal cycling in dune slacks. Plant and Soil, 396, 411–420. 10.1007/s11104-015-2628-9

[ece36743-bib-0059] Murray, B. D. , Webster, C. R. , & Bump, J. K. (2013). Broadening the ecological context of ungulate–ecosystem interactions: The importance of space, seasonality, and nitrogen. Ecology, 94, 1317–1326. 10.1890/12-1582.1 23923495

[ece36743-bib-0060] Murray, T. R. , Frank, D. A. , & Gehring, C. A. (2010). Ungulate and topographic control of arbuscular mycorrhizal fungal spore community composition in a temperate grassland. Ecology, 91, 815–827. 10.1890/09-0209.1 20426339

[ece36743-bib-0061] Neff, J. C. , Reynolds, R. , Sanford, R. L. Jr , Fernandez, D. , & Lamothe, P. (2006). Controls of bedrock geochemistry on soil and plants nutrients in Southeastern Utah. Ecosystems, 9, 879–893.

[ece36743-bib-0062] Pastor, J. , Dewey, B. , Naiman, R. J. , McInnes, P. , & Cohen, Y. (1993). Moose browsing and soil fertility in the boreal forests of Isle Royale National Park. Ecology, 74, 467–480. 10.2307/1939308

[ece36743-bib-0063] Pastor, J. , & Naiman, R. J. (1992). Selective foraging and ecosystem processes in boreal forests. American Naturalist, 139, 690–705. 10.1086/285353

[ece36743-bib-0064] Post, W. M. , & Pastor, J. (1988). An individual‐based forest ecosystems model for projecting forest response to nutrient cycling and climate changes. United States Department of Energy, Office of Scientific and Technical Information.

[ece36743-bib-0065] Pupin, B. , da Silva Freddi, O. , & Nahas, E. (2008). Microbial alterations of the soil influenced by induced compaction. Revista Brasileira de Ciencia do Solo, 33(5), 1207–1213. 10.1590/S0100-06832009000500014

[ece36743-bib-0066] Relva, M. A. , Castan, E. , & Mazzarino, M. J. (2014). Litter and soil properties are not altered by invasive deer browsing in forests of NW Patagonia. Acta Oecologia, 54, 45–50. 10.1016/j.actao.2012.12.006

[ece36743-bib-0068] Rhodes, A. C. , Anderson, V. , & St. Clair, S. B. (2017). Ungulate herbivory alters leaf functional traits and recruitment of regenerating aspen. Tree Physiology, 37, 402–413. 28338915 10.1093/treephys/tpx015

[ece36743-bib-0069] Ricca, M. A. , Miles, A. K. , Van Vuren, D. H. , & Eviner, V. T. (2016). Impacts of introduced *Rangifer* on ecosystem processes of maritime tundra on subarctic islands. Ecosphere, 7, e01219.

[ece36743-bib-0070] Riginos, C. , & Grace, J. B. (2008). Savanna tree density, herbivores, and the herbaceous community: Bottom‐up vs. top‐down effects. Ecology, 89, 2228–2238. 18724733 10.1890/07-1250.1

[ece36743-bib-0071] Ritchie, M. E. , Tilman, D. , & Knops, J. M. H. (1998). Herbivore effects on plant and nitrogen dynamics in oak savanna. Ecology, 79, 165–177. 10.1890/0012-9658(1998)079[0165:HEOPAN]2.0.CO;2

[ece36743-bib-0072] Ruess, R. W. , & McNaughton, S. J. (1987). Grazing and the dynamics of nutrient and energy regulated microbial processes in the Serengeti grasslands. Oikos, 49, 101–110. 10.2307/3565559

[ece36743-bib-0073] Schonecker, K. A. , Singer, F. J. , Zeigenfuss, L. C. , Binkley, D. , & Menezes, R. (2004). Effects of elk herbivory on vegetation and nitrogen processes. Journal of Wildlife Management, 68, 837–849. 10.2193/0022-541X(2004)068[0837:EOEHOV]2.0.CO;2

[ece36743-bib-0074] Schrama, M. , Ciska Veen, G. F. , Bakker, E. S. L. , Ruifrok, J. L. , Bakker, J. P. , & Olff, H. (2013). An integrated perspective to explain nitrogen mineralization in grazed ecosystems. Perspectives in Plant Ecology, Evolution and Systematics, 15, 32–44.

[ece36743-bib-0075] Schrama, M. , Heijning, P. , Bakker, J. P. , van Wijnen, H. J. , Berg, M. P. , & Olff, H. (2013). Herbivore trampling as an alternative pathway for explaining differences in nitrogen mineralization in moist grasslands. Oecologia, 172, 231–243. 10.1007/s00442-012-2484-8 23271034

[ece36743-bib-0100] Seagle, S. W. (2003). Can ungulates foraging in a multiple‐use landscape alter forest nitrogen budgets?. Oikos, 103(1), 230–234.

[ece36743-bib-0076] Senft, R. L. , Coughenour, M. B. , Bailey, D. W. , Rittenhouse, L. R. , Sala, O. E. , & Swift, D. M. (1987). Large herbivore foraging and ecological hierarchies. BioScience, 37, 789–799. 10.2307/1310545

[ece36743-bib-0077] Singer, F. J. , & Schoenecker, K. A. (2003). Do ungulates accelerate or decelerate nitrogen cycling? Forest Ecology and Management, 181, 189–204. 10.1016/S0378-1127(03)00133-6

[ece36743-bib-0078] Sitters, J. , & Olde Venterink, H. (2015). The need for a novel integrative theory on feedbacks between herbivores, plants and soil nutrient cycling. Plant and Soil, 396, 421–426. 10.1007/s11104-015-2679-y

[ece36743-bib-0079] Sitters, J. , te Beest, M. , Cherif, M. M. , Giesler, R. , & Olofsson, J. (2017). Interactive effects between reindeer and habitat fertility drive soil nutrient availability in arctic tundra. Ecosystems, 20, 1266–1277.

[ece36743-bib-0081] Stark, S. , & Grellmann, D. (2002). Soil microbial responses to herbivory in an arctic tundra heath at two levels of nutrient availability. Ecology, 83, 2736–2744. 10.1890/0012-9658(2002)083[2736:SMRTHI]2.0.CO;2

[ece36743-bib-0082] Steffens, M. , Kölbl, A. , Totsche, K. U. , & Kögel‐Knabner, I. (2008). Grazing effects on soil chemical and physical properties in a semiarid steppe of Inner Mongolia (PR China). Geoderma, 143, 63–72. 10.1016/j.geoderma.2007.09.004

[ece36743-bib-0085] Tan, X. , Chang, S. X. , & Kabzems, R. (2008). Soil compaction and forest floor removal reduced microbial biomass and enzyme activities in a boreal aspen forest soil. Biology and Fertility of Soils, 44, 471–479. 10.1007/s00374-007-0229-3

[ece36743-bib-0086] Teague, R. , & Barnes, M. (1997). Grazing management that regenerates ecosystem function and grazing land livelihoods. African Journal of Range and Forage Science, 34, 77–86. 10.2989/10220119.2017.1334706

[ece36743-bib-0087] Thrash, I. (1997). Infiltration rate of soil around drinking troughs in the Kruger National Park, South Africa. Journal of Arid Environments, 35, 617–625. 10.1006/jare.1996.0227

[ece36743-bib-0088] Torbert, H. A. , & Wood, C. W. (1992). Effects of soil compaction and water‐filled pore space on soil microbial activity and N losses. Communications in Soil Science and Plant Analysis, 23, 1321–1331. 10.1080/00103629209368668

[ece36743-bib-0090] Vaieretti, M. V. , Cingolani, A. M. , Pérez Harguindeguy, N. , & Cabido, M. (2013). Effects of differential grazing on decomposition rate and nitrogen availability in a productive mountain grassland. Plant and Soil, 371, 675–691. 10.1007/s11104-013-1831-9

[ece36743-bib-0091] Vallentine, J. F. (1990). Grazing management (p. 533). San Diego, CA: Academic Press.

[ece36743-bib-0092] Van der Wal, R. , van Lieshout, S. M. J. , & Loonen, M. J. J. E. (2001). Herbivore impact on moss depth, soil temperature and arctic plant growth. Polar Biology, 24, 29–32. 10.1007/s003000000170

[ece36743-bib-0093] Van Haveren, B. P. (1983). Soil bulk density as influenced by grazing intensity and soil type on a shortgrass prairie type. Journal of Range Management, 36, 586–588.

[ece36743-bib-0094] Veldhuis, M. P. , Howison, R. A. , Rienk, W. , Fokkema, R. W. , Tielens, E. , & Olff, H. (2014). A novel mechanism for grazing lawn formation: Large herbivore‐induced modification of the plant‐soil water balance. Journal of Ecology, 102, 1506–1517. 10.1111/1365-2745.12322

[ece36743-bib-0095] Verchot, L. V. , Groffman, P. M. , & Frank, D. A. (2002). Landscape versus ungulate control of gross mineralization and gross nitrification in semi‐arid grasslands of Yellowstone National Park. Soil Biology and Biochemistry, 34, 1691–1699. 10.1016/S0038-0717(02)00155-4

[ece36743-bib-0097] Wardle, D. A. , Bardgett, R. D. , Klironomos, J. N. , Setälä, H. , van der Putten, W. H. , & Wall, D. H. (2004). Ecological linkages between aboveground and belowground biota. Science, 304, 1629–1633. 10.1126/science.1094875 15192218

[ece36743-bib-0098] Young, T. P. , Palmer, T. M. , & Gadd, M. E. (2005). Competition and compensation among cattle, zebras, and elephants in a semi‐arid savanna in Laikipia, Kenya. Biological Conservation, 122, 351–359. 10.1016/j.biocon.2004.08.007

