# Supplementary material for: Context‐dependent effects of a reintroduced ungulate on soil properties are driven by soil texture, moisture, and herbivore activity
Source: Ecol Evol. 2020 Sep 7;10(19):10858–71. doi: 10.1002/ece3.6743 (PMC7548165; doi:10.1002/ece3.6743)
Supplement: Supplementary file 1 — Fig S1 [file ECE3-10-10858-s001.pptx]

## Slide 1
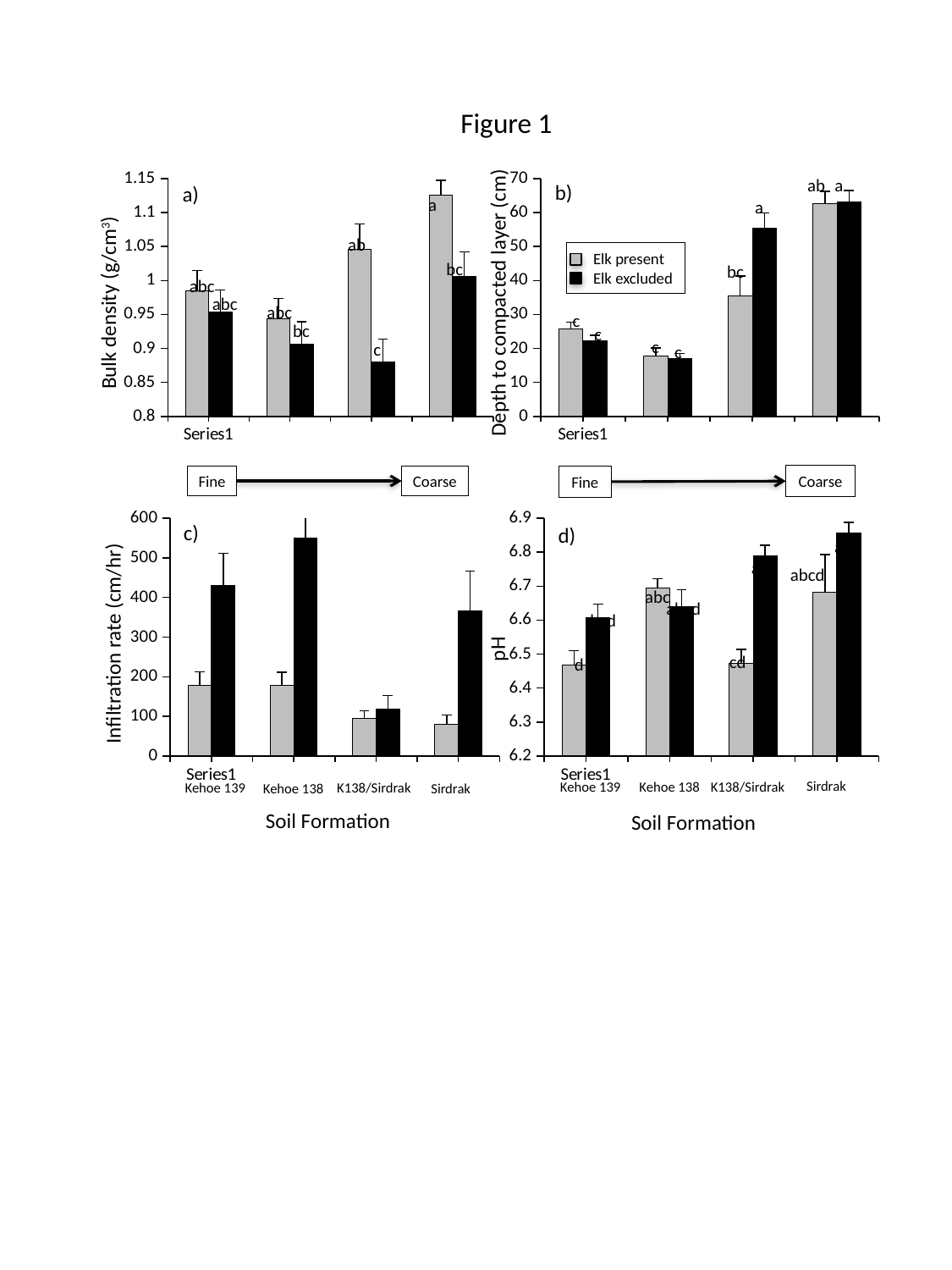

Figure 1
### Chart
| Category | Elk present | Elk excluded |
|---|---|---|
| | 0.9847523186 | 0.9538523414 |
| | 0.9440376704 | 0.9068692708 |
| | 1.0452812058 | 0.8805501095 |
| | 1.1253981364 | 1.0054535247 |
### Chart
| Category | Elk present | Elk excluded |
|---|---|---|
| | 25.7528 | 22.225 |
| | 17.85 | 17.003 |
| | 35.56 | 55.316 |
| | 62.654 | 63.218 |ab
a
b)
a)
a
a
ab
 Elk present
 Elk excluded
bc
bc
abc
Depth to compacted layer (cm)
Bulk density (g/cm3)
abc
abc
c
bc
c
c
c
c
Coarse
Fine
Coarse
Fine
### Chart
| Category | Elk present | Elk excluded |
|---|---|---|
| | 178.70060742 | 431.17448042 |
| | 178.5490557799999 | 549.81811498 |
| | 95.131653566 | 118.51226244 |
| | 79.317499411 | 366.16503761 |
### Chart
| Category | Elk present | Elk excluded |
|---|---|---|
| | 6.4666666667 | 6.6083333333 |
| | 6.694444444399984 | 6.638888888899986 |
| | 6.4722222222 | 6.7888888889 |
| | 6.6833333333 | 6.855555555599977 |c)
d)
a
ab
abcd
abc
abcd
bcd
Infiltration rate (cm/hr)
pH
cd
d
Sirdrak
K138/Sirdrak
Kehoe 138
Kehoe 139
K138/Sirdrak
Kehoe 139
Kehoe 138
Sirdrak
Soil Formation
Soil Formation

## Slide 2
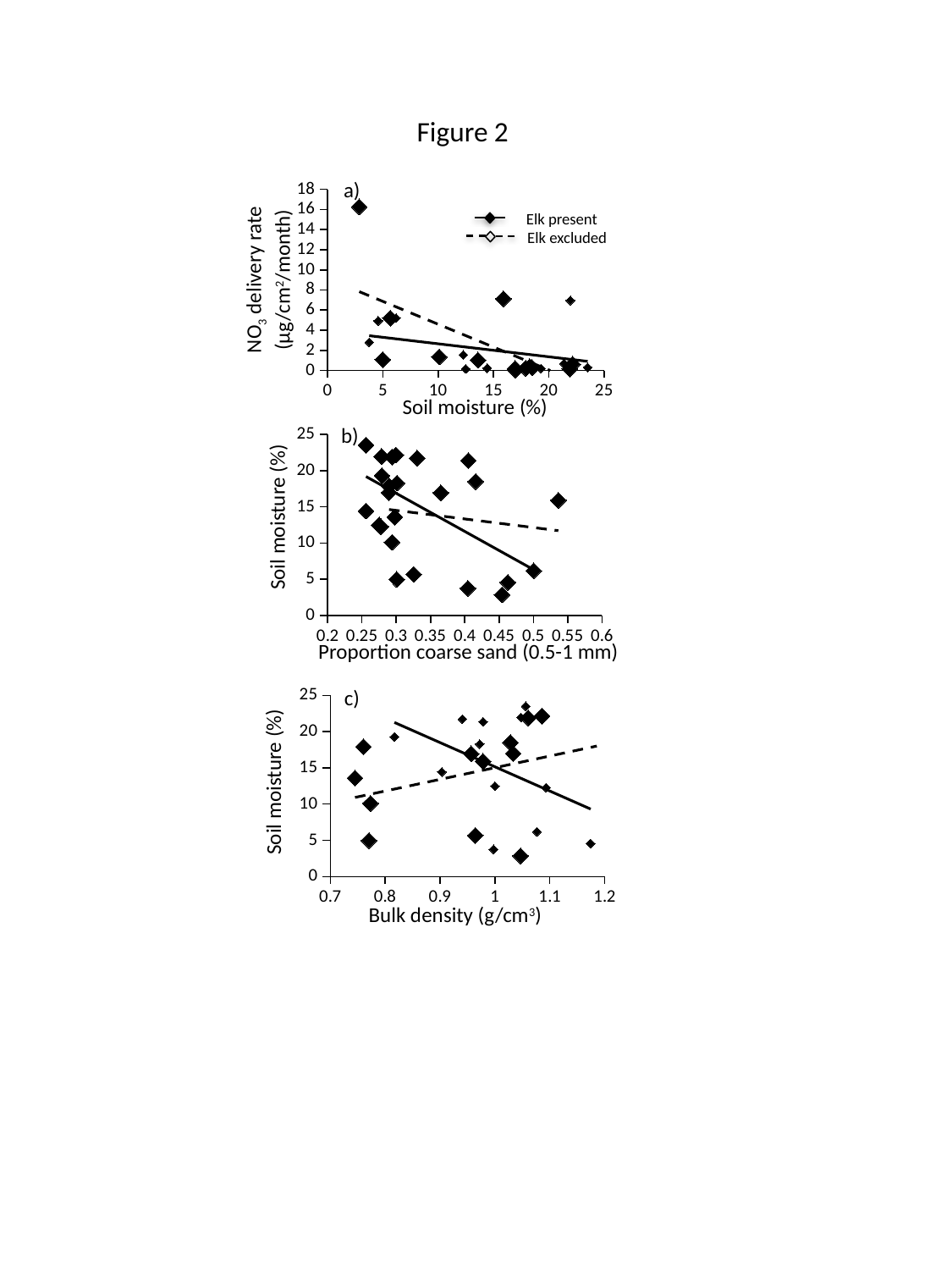

Figure 2
a)
### Chart
| Category | | |
|---|---|---| Elk present
 Elk excluded
NO3 delivery rate
(μg/cm2/month)
Soil moisture (%)
b)
### Chart
| Category | | |
|---|---|---|Soil moisture (%)
Proportion coarse sand (0.5-1 mm)
c)
### Chart
| Category | | |
|---|---|---|Soil moisture (%)
Bulk density (g/cm3)

## Slide 3
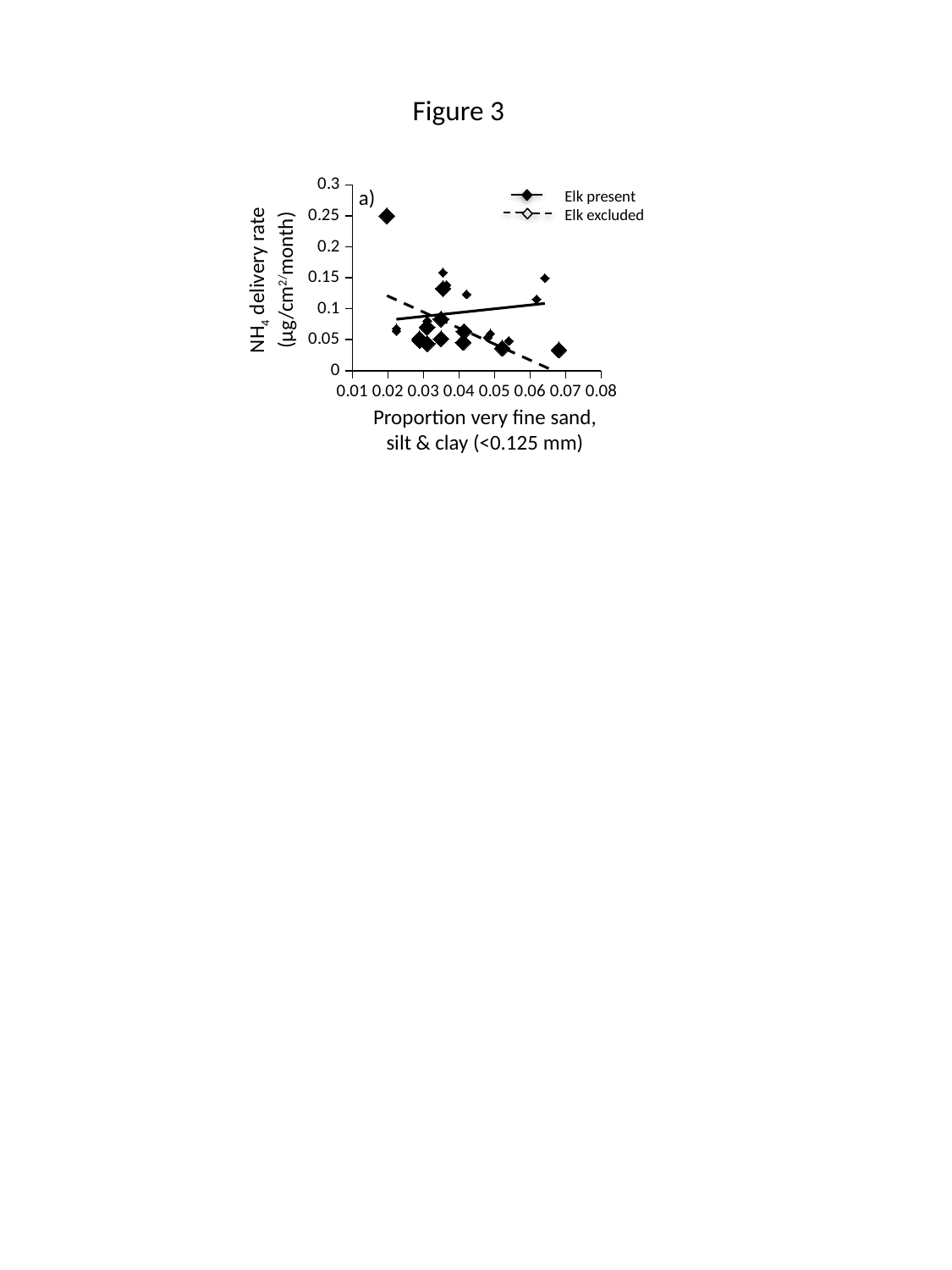

Figure 3
### Chart
| Category | | |
|---|---|---|a)
 Elk present
 Elk excluded
NH4 delivery rate
(μg/cm2/month)
Proportion very fine sand, silt & clay (<0.125 mm)

## Slide 4
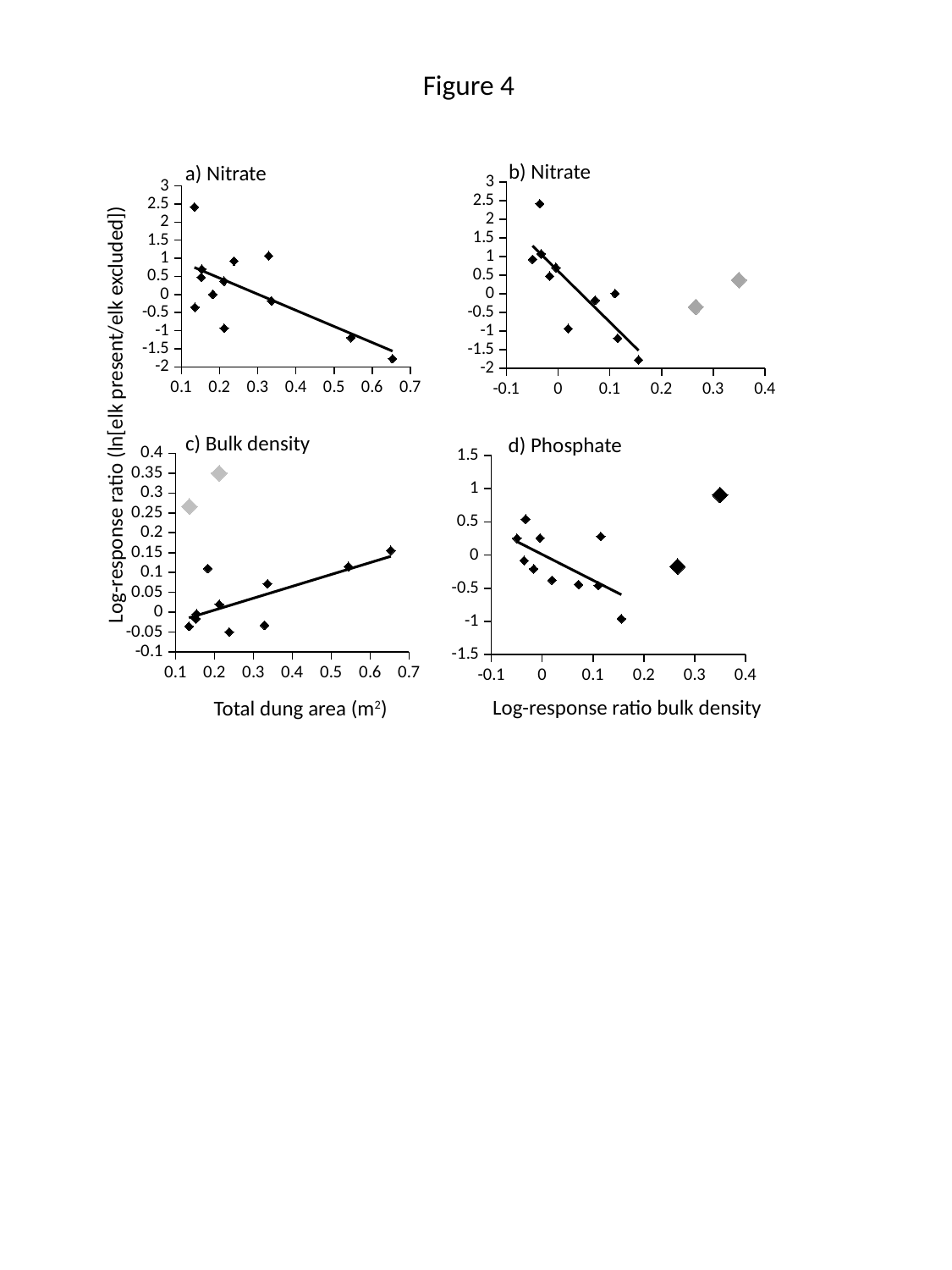

Figure 4
b) Nitrate
### Chart
| Category | LRRNO3 |
|---|---|a) Nitrate
### Chart
| Category | | |
|---|---|---|Log-response ratio (ln[elk present/elk excluded])
c) Bulk density
d) Phosphate
### Chart
| Category | LRR Phos | |
|---|---|---|
### Chart
| Category | | |
|---|---|---|Log-response ratio bulk density
Total dung area (m2)

## Slide 5
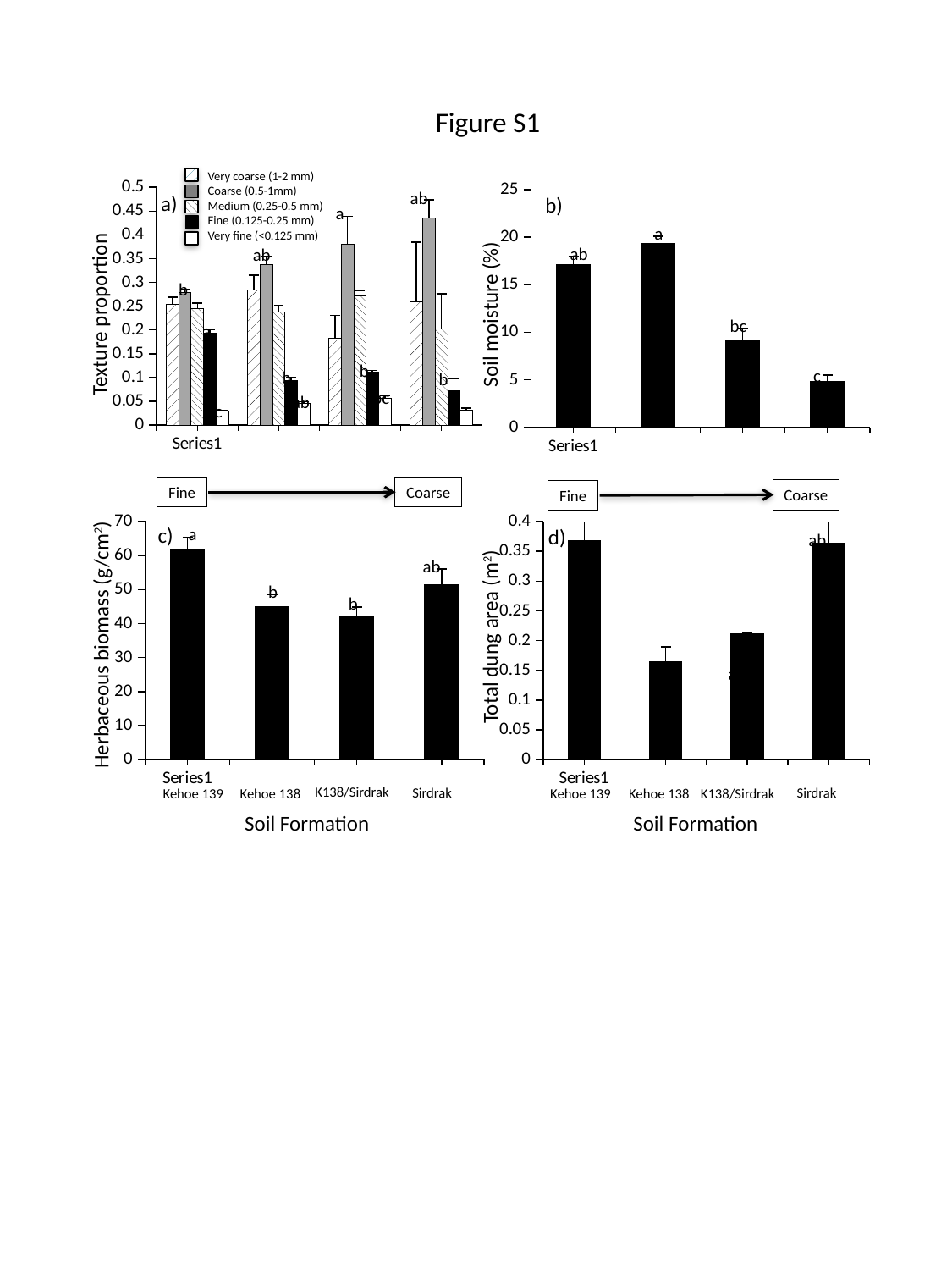

Figure S1
 Very coarse (1-2 mm)
 Coarse (0.5-1mm)
 Medium (0.25-0.5 mm)
 Fine (0.125-0.25 mm)
 Very fine (<0.125 mm)
### Chart
| Category | V. coarse sand | Coarse sand | Med. Sand | Fine sand | V. sand/silt/clay |
|---|---|---|---|---|---|
| | 0.25425 | 0.2791850631 | 0.2452332716 | 0.1924492753 | 0.0288272011 |
| | 0.284625 | 0.3367365339 | 0.2386235823 | 0.0940230168 | 0.0461498141 |
| | 0.18325 | 0.3797900505 | 0.2711118055 | 0.1108976055 | 0.0556192963 |
| | 0.25925 | 0.4357282663 | 0.2015475933 | 0.0717713225 | 0.031759882 |
### Chart
| Category | VM (%) |
|---|---|
| | 17.0825 |
| | 19.3225 |
| | 9.229999999999999 |
| | 4.8322580645 |ab
a)
b)
a
a
ab
ab
b
Texture proportion
 Soil moisture (%)
bc
a
b
c
b
b
bc
ab
a
c
Fine
Coarse
Coarse
Fine
### Chart
| Category | Biomass |
|---|---|
| | 61.87727777799999 |
| | 45.074902778 |
| | 41.991222222 |
| | 51.523628571 |
### Chart
| Category | Dung area |
|---|---|
| | 0.368 |
| | 0.1649 |
| | 0.2122 |
| | 0.3635 |c)
a
d)
ab
ab
a
b
b
Herbaceous biomass (g/cm2)
Total dung area (m2)
ab
b
K138/Sirdrak
Sirdrak
Sirdrak
Kehoe 139
Kehoe 138
Kehoe 139
Kehoe 138
K138/Sirdrak
Soil Formation
Soil Formation
