# Supplementary material for: Context‐dependent effects of a reintroduced ungulate on soil properties are driven by soil texture, moisture, and herbivore activity
Source: Ecol Evol. 2020 Sep 7;10(19):10858–71. doi: 10.1002/ece3.6743 (PMC7548165; doi:10.1002/ece3.6743)
Supplement: Supplementary file 2 — Table S1 [file ECE3-10-10858-s002.docx]

**Table S1.** Correlation coefficients for physical soil variables including very coarse sand (1-2 mm), coarse sand (0.5-1 mm), medium sand (0.25-0.5 mm), fine sand (0.125-0.25 mm), very fine material (sand, silt and clay) (<0.125 mm), volumetric moisture, bulk density, infiltration rate, penetration resistance, and pH.

|  | V. coarse sand | Coarse sand | Medium sand | Fine  sand | V. fine material | Volumetric  Moisture | Bulk density | Infiltration rate | Penetration resistance | pH |
| --- | --- | --- | --- | --- | --- | --- | --- | --- | --- | --- |
| V. coarse sand | 1.0000 | -0.4522 | -0.8370 | -0.3511 | -0.0545 | 0.1620 | -0.0944 | -0.0858 | -0.1355 | -0.0046 |
| Coarse sand | -0.4522 | 1.0000 | -0.0212 | -0.5430 | 0.0333 | -0.4287 | 0.0221 | 0.3765 | 0.510 | 0.2472 |
| Medium sand | -0.8370 | -0.0212 | 1.0000 | 0.5006 | 0.1938 | 0.0360 | 0.0393 | -0.0879 | -0.1327 | -0.0861 |
| Fine  sand | -0.3511 | -0.5430 | 0.5006 | 1.0000 | -0.3927 | 0.2785 | -0.0164 | -0.2466 | -0.2940 | -0.2612 |
| V. fine material | -0.0545 | 0.0333 | 0.1938 | -0.3927 | 1.0000 | -0.0822 | -0.0421 | -0.0444 | -0.0934 | 0.0049 |
| Volumetric  Moisture | 0.1620 | -0.4287 | 0.0360 | 0.2785 | -0.0822 | 1.0000 | 0.1184 | -0.3263 | 0.1184 | -0.0898 |
| Bulk density | -0.0944 | 0.0221 | 0.0393 | -0.0164 | -0.0421 | 0.1184 | 1.0000 | 0.1646 | 0.1049 | 0.0470 |
| Infiltration rate | -0.0858 | 0.3765 | -0.0879 | -0.2466 | -0.0444 | -0.3263 | 0.1646 | 1.0000 | 0.4161 | 0.1550 |
| Penetration resistance | -0.1355 | 0.510 | -0.1327 | -0.2940 | -0.0934 | 0.1184 | 0.1049 | 0.4161 | 1.0000 | 0.110 |
| pH | -0.0046 | 0.2472 | -0.0861 | -0.2612 | 0.0049 | -0.0898 | 0.0470 | 0.1550 | 0.110 | 1.0000 |
