# Supplementary material for: Context‐dependent effects of a reintroduced ungulate on soil properties are driven by soil texture, moisture, and herbivore activity
Source: Ecol Evol. 2020 Sep 7;10(19):10858–71. doi: 10.1002/ece3.6743 (PMC7548165; doi:10.1002/ece3.6743)
Supplement: Supplementary file 3 — Fig S1‐cap [file ECE3-10-10858-s003.docx]

**Figure S1.** Mean +/- 1 SE texture proportion (a), %soil moisture (b), dry aboveground plant biomass taken during peak biomass (c), and mean dung area in 36 m x 36 m plot (d) as a function of soil formation (Kehoe 138, Kehoe 139, Sirdrak Sand, and mixed Kehoe 138/Sirdrak Sand. Graphs a, b, and c represent averages of inside and outside exclosures. Measurements of elk dung area for graph d are from outside exclosures. Letters above bars correspond to the results from Tukey multiple comparison tests. * Herbaceous biomass only. Does not include woody biomass.
